# Powerful Avidity with a Limited Valency for Virus-Attachment Blockers on DC-SIGN: Combining Chelation and Statistical Rebinding with Structural Plasticity of the Receptor

**DOI:** 10.1021/acscentsci.2c01136

**Published:** 2023-02-20

**Authors:** Vanessa Porkolab, Martin Lepšík, Stefania Ordanini, Alexander St John, Aline Le Roy, Michel Thépaut, Emanuele Paci, Christine Ebel, Anna Bernardi, Franck Fieschi

**Affiliations:** † Univ. Grenoble Alpes, CNRS, CEA, Institut de Biologie Structurale, 38000 Grenoble, France; ¶ Univ. Grenoble Alpes, CNRS, CERMAV, 38000 Grenoble, France; ■ Institute of Organic Chemistry and Biochemistry, Czech Academy of Sciences, Flemingovo nam. 2, Prague 6, 166 10, Czechia; § Universita’ degli Studi di Milano, Dipartimento di Chimica, via Golgi 19, 20133, Milano, Italy; ∥ Astbury Centre & School of Molecular and Cellular Biology, University of Leeds, Leeds, LS2 9JT, United Kingdom; ⊥ Department of Physics and Astronomy “Augusto Righi”, University of Bologna, Via Zamboni, 33, 40126, Bologna, Italy; ‡ Institut Universitaire de France (IUF), 1 rue Descartes, 75231 Paris, France

## Abstract

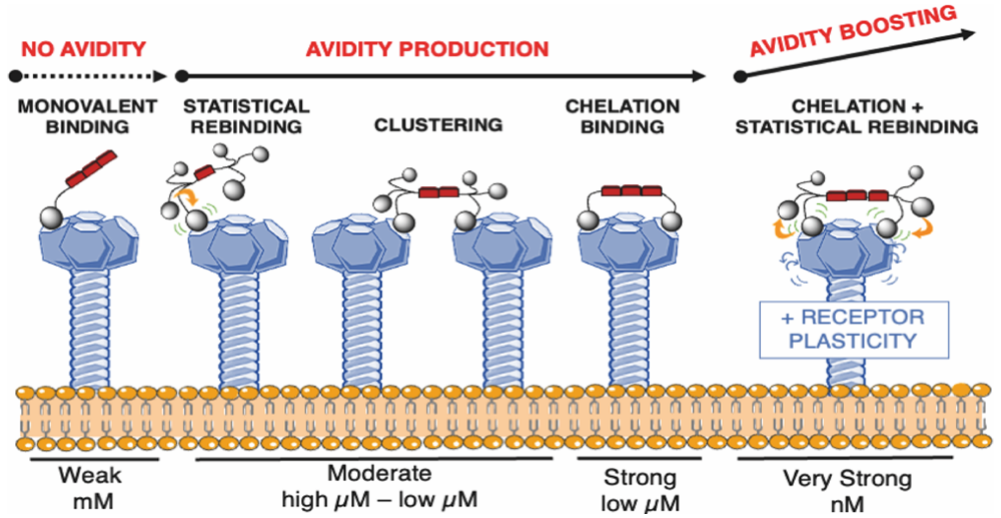

The C-type lectin receptor DC-SIGN has been highlighted as the coreceptor for the spike protein of the SARS-CoV-2 virus. A multivalent glycomimetic ligand, Polyman26, has been found to inhibit DC-SIGN-dependent trans-infection of SARS-CoV-2. The molecular details underlying avidity generation in such systems remain poorly characterized. In an effort to dissect the contribution of the known multivalent effects — chelation, clustering, and statistical rebinding — we studied a series of dendrimer constructs related to Polyman26 with a rod core rationally designed to engage simultaneously two binding sites of the tetrameric DC-SIGN. Binding properties of these compounds have been studied with a range of biophysical techniques, including recently developed surface plasmon resonance oriented-surface methodology. Using molecular modeling we addressed, for the first time, the impact of the carbohydrate recognition domains’ flexibility of the DC-SIGN tetramer on the compounds’ avidity. We were able to gain deeper insight into the role of different binding modes, which in combination produce a construct with a nanomolar affinity despite a limited valency. This multifaceted experimental–theoretical approach provides detailed understanding of multivalent ligand/multimeric protein interactions which can lead to future predictions. This work opens the way to the development of new virus attachment blockers adapted to different C-type lectin receptors of viruses.

## Abstract 2

Interaction studies in solution and on oriented surfaces, coupled with molecular dynamics, elucidate avidity mechanisms. A new paradigm to optimize multivalent ligands targeting oligomeric receptors.

## Introduction

Multivalency is commonly used by Nature to achieve high avidities of larger carbohydrates toward their oligomeric lectin receptors. We study this phenomenon in C-type lectin receptors (CLRs) in which glycan binding occurs via Ca^2+^ in the carbohydrate recognition domains (CRDs). Among CLRs, the dendritic cell membrane receptor DC-SIGN plays numerous roles in human immune system^1^ interacting with specific carbohydrate structures (fucose, Lewis-type, and high-mannose moieties) expressed on self-glycoproteins^2^ and pathogens.^3−7^

**Figure 1:**
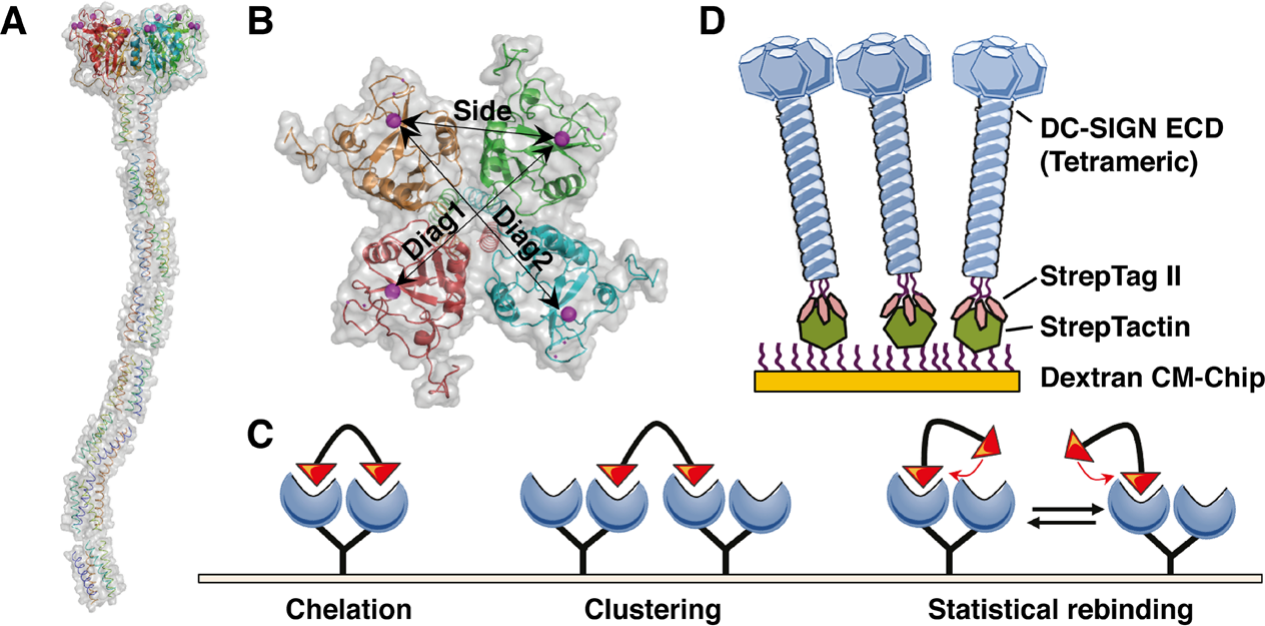
DC-SIGN extracellular domain as a tetrameric binding platform. A. Side view, combination of CRD and neck structures fitted into SAXS envelope of whole ECD.^9^ B. Top view of tetrameric head. For clarity only the Ca^2+^ ions of the carbohydrate binding site are represented (magenta). The distance between 2 Ca^2+^ binding sites spans a length of ∼39 Å between 2 adjacent sites (*Side*), ∼52 Å and ∼60 Å for 2 respective opposite sites (*Diag1* and *Diag2*; Table 3). C. Theoretical multivalent binding modes between a simplified bivalent ligand and a protein receptor with two binding sites (red triangle represents the “spearhead”). D. The DC-SIGN oriented surface setup, via a *Strep*Tag/*Strep*Tactin coupling, for SPR measurements. It mimics the presentation and accessibility of binding sites on the cell surface and thus allows one to observe the resultant of the different binding modes simultaneously.^20^

The extracellular domain (ECD) of DC-SIGN oligomerizes as a homotetramer via a coiled-coil neck region and carries CRDs at the C-terminus of each monomer in a square-like arrangement (Figure 1A,B).^8,9^ With the aim of preventing pathogen binding or modulating the immune responses brought about by DC-SIGN, numerous carbohydrate-based ligands were designed which bound to monomeric CRDs.^10−13^ These efforts have led to the development of optimized glycomimetics with affinities in the μM range.^14−16^ Enhancing the interaction strength via multivalent display of these “glycomimetic spearheads” led to improving binding avidities to the nM range.^17−20^

Multivalent effects can be of different types — statistical rebinding, clustering, and chelation (Figure 1C). Over the past 20 years, researchers have shown an increased interest in synthesizing multivalent ligands, especially for lectin binding,^21,22^ which have often been built in a relatively unspecific fashion, privileging high-number valencies and relatively uncontrolled, flexible scaffolds. This type of design maximizes statistical rebinding effects and allows protein clustering in solution, while the occasional use of very large high-valency scaffolds^23,24^ also capitalizes on the possibility of binding simultaneously to more than one CRD of the same (chelating) or different (clustering) lectin oligomer. However, the loss of flexibility upon binding generates unfavorable entropy.^25,26^ An alternative strategy is thus to use smaller constructs of limited valency, which requires a careful design of the scaffold to meet the requirements for linker length and properties.^27−31^ It has been repeatedly shown that rigid linkers, unless exactly matched to the size of the receptor, are often too unforgiving of minor design inaccuracies and possibly not well-suited to the intrinsically flexible nature of proteins. Thus, an appropriate balance between rigidity and flexibility of the linker must be struck in order to maximize the possibility of binding events to occur productively and simultaneously in two or more protein binding sites, while minimizing the entropy losses. When this can occur, the level of avidity generated can yield several orders of magnitude as compared to that obtained by statistical rebinding effects only.^27^

We have previously succeeded in this task using a modular design that includes a linear rigid “rod-like” spacer of controllable length to connect two flexibles trivalent dendrons, each carrying three glycomimetic DC-SIGN ligands. The activity of the resulting constructs, as measured in DC-SIGN competition experiments and in a cellular model of DC-SIGN mediated HIV infection, was shown to depend on the length of the rod, the valency, and the affinity of the monovalent ligand.^19,32^ This study led to dendrimer **3.6** (Table 1, also called Polyman26), a nanomolar inhibitor of DC-SIGN mediated HIV transmission^19^ which is internalized by dendritic cells and induces β-chemokine and pro-inflammatory cytokine production.^11^ More recently, Polyman26 also showed its capacity to inhibit, *in vitro* and in a cellular assay, the DC-SIGN-dependent trans-infection of the SARS-CoV-2 pseudovirus as well as authentic SARS-CoV-2 virus.^7^

**Table 1:**
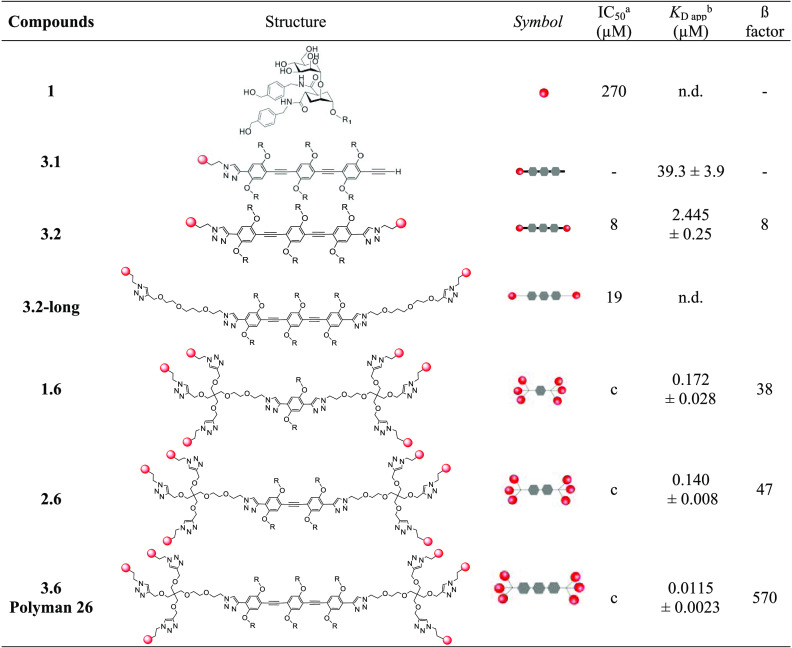
Schematic Representation of Rigid Rod-Based Dendrimers and the *K*_Dapp_ Values Obtained by SPR on a DC-SIGN ECD Oriented Surface^d^

d IC_50_ values obtained in SPR inhibition experiments (from ref (19)) are included for comparison. Valency-corrected enhancement factors^13^ (β) as compared to the modified pseudo-dimannoside spearhead **1** are shown. R = CH_2_CH_2_OCH_2_CH_2_OH and R_1_ = CH_2_CH_2_N_3_.

a From SPR inhibition study (competition experiment) (ref (19)).

b from SPR direct interaction measurement on a DC-SIGN ECD oriented surface (this work).

c IC_50_ values reported in ref (19) for these compounds were not significant (SPR competition experiment) because the lower limit of the test was reached.

Here we collect new interaction data obtained with several biophysical techniques for a series of rod-based constructs, including appropriate controls, and apply molecular modeling including CRDs flexibility, in order to establish a detailed structure–activity relationship analysis of multivalent effects. Due to the multivalency in both the DC-SIGN tetramer and the hexavalent rod-like dendrimers, unraveling the complex and dynamic interaction required the use of several complementary techniques, which notably include a surface plasmon resonance (SPR) oriented-DC-SIGN ECD surface methodology (Figure 1D) that we have recently developed.^20^ As for the theoretical methods, we used molecular dynamics of the whole DC-SIGN tetramer ECD to assess the variability of inter-CRD distances, which is rarely addressed in multivalent interaction studies.^33^ Altogether, these results allowed us to gain structural and thermodynamic insights into the generation of avidity in the DC-SIGN tetramer/multivalent construct system and to define a new strategy for future development of other virus attachment blockers toward other C-type lectin receptors.

## Results and Discussion

The full set of ligands analyzed is collected in Table 1. They all carry the modified pseudodimannoside **1** as the active spearhead. Dendrimers **1.6**, **2.6**, and **3.6** are all hexavalent and differ by the length of the spacer, which goes from one to three aromatic units (as indicated by the first digit in their numbering). Compounds **3.1**, **3.2**, **3.2-long**, and **3.6** share the extended (3 units) rigid core, which has a length predicted previously to span two neighboring binding sites (∼39 Å) (Figure 1B).^9^ As indicated by the second digit in their numbering, they are mono-, di-, or hexa-valent, respectively. Compound **3.6** corresponds to the previously shown optimum^19^ of both valency and central core length.

**Figure 2:**
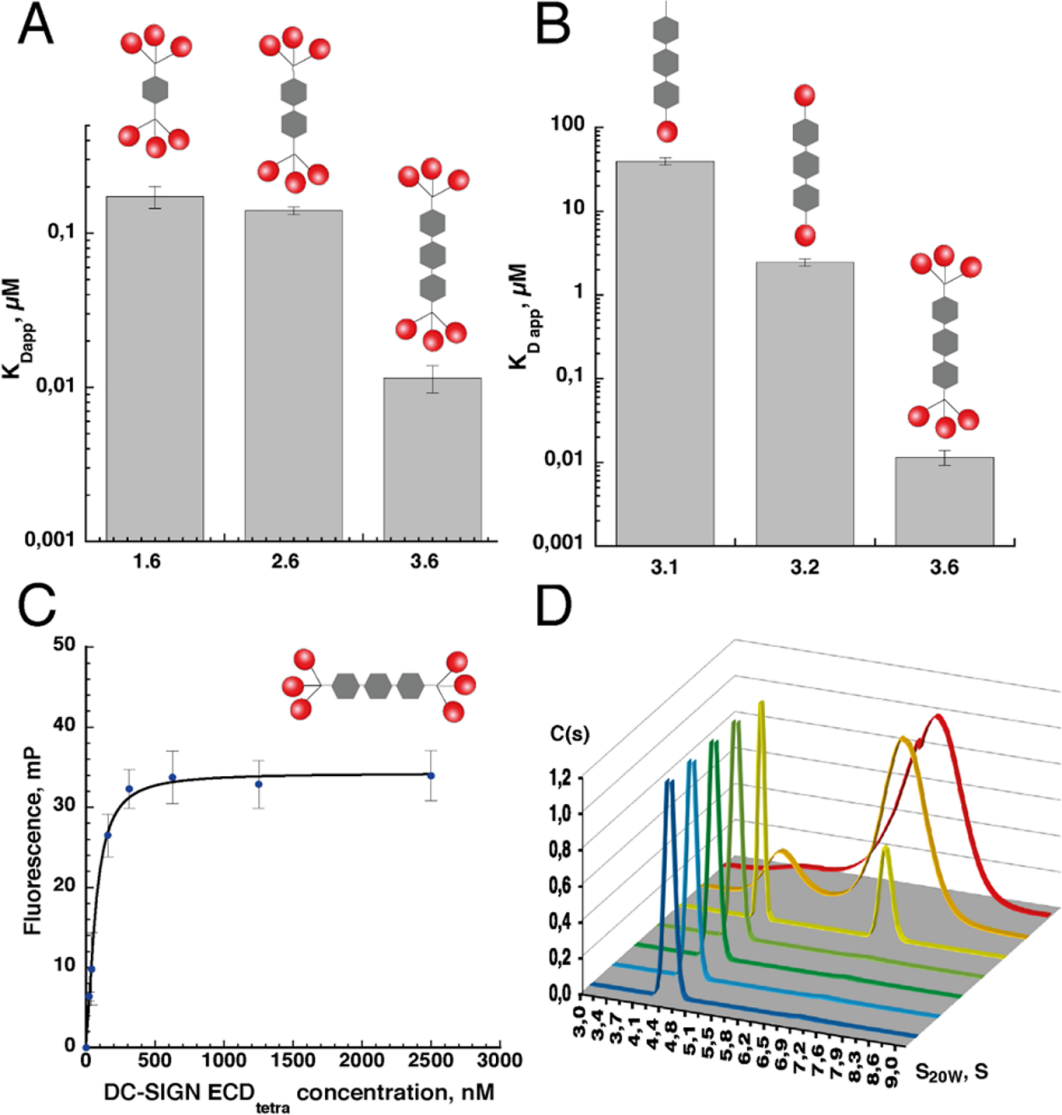
Binding properties of compound **3.6**. A. Impact of rod core length on *K*_Dapp_ values obtained by compounds titration over a DC-SIGN S-ECD surface. B. Impact of valency on *K*_Dapp_ values obtained by compounds titration over a DC-SIGN S-ECD surface. C. Direct binding assay using fluorescence polarization equilibrium measurements. Binding of compound **3.6** (400 nM) at increasing concentrations of DC-SIGN ECD tetramer (from 2.5 μM, serial dilution ×2) are shown. D. Sedimentation velocity experiments of DC-SIGN tetramer alone (4 μM, black line) and with increasing concentrations of compound **3.6** (0.005, 0.05, 0.5, 2, 4, and 6 μM, from dark blue to red lines).

Previously, these glycodendrimers were tested for their ability to inhibit binding of DC-SIGN ECD to immobilized BSA-Man or gp120 surfaces.^20^ However, we showed that in some cases, this SPR competition assay setting shows clear limitations as a tool for affinity determination,^20^ due to the underestimation of surface-avidity phenomenon acting as a leading contributor of multivalent binding. In addition, in the case at hand, the lower limit of the inhibition assay sensitivity (affinity of the reporter interaction itself) was reached for concentrations of the ligand in the low μM range. Thus, the system could be used to compare monovalent and divalent compounds (Table 1, compounds **1**, **3.1**, **3.2**, **3.2-long**, and others in ref (19) but failed to afford meaningful information for compounds **1.6**, **2.6**, and **3.6**. In order to investigate the mechanism of these complex interactions, we used the recently developed SPR analysis using DC-SIGN ECD-oriented surface^20^ as the most suitable approach. It mimics DC-SIGN presentation at the plasma membrane, with all CRDs accessible, as well as the multivalent binding potential of the cell surface.^20^ For the experiment, increasing concentrations of glycodendrimers were injected over the oriented surface. Sensorgrams are shown in Figure S1, *K*_Dapp_ values are summarized in Table 1. Comparing the hexavalent series, reported in Figure 2A, the same range of affinity was observed between compounds **1.6** and **2.6**, with an apparent *K*_D_ (*K*_Dapp_) of 0.17 and 0.14 μM respectively. On the contrary, an improvement by a factor of 10 was found for compound **3.6** with an affinity in the nM range (*K*_Dapp_ = 11.5 nM), which compares well with the antiviral activity measured for this molecule in HIV and SARS-CoV-2 trans-infection assay.^7,19^ This striking result confirms that the length of the rigid spacer is critical to improve binding affinity with DC-SIGN.

Figure 2B shows the role of valency (and partly of the flexible extensions in **3.6**) for dendrimers sharing the same rod size. Remarkably, the *K*_D_ values of ligands **3.1** (39.3 ± 3.9 μM), **3.2** (2.4 ± 0.25 μM), and **3.6** (11.5 ± 2.3 nM) show a regular increase of the affinity by 1 order of magnitude as the valency increases from 1 to 2 and by 2 orders of magnitude as the valency increases from 2 to 6 and more flexible extensions are added. There is a 6-fold factor between the IC_50_ and *K*_Dapp_ affinity measured for the spearhead **1** and for **3.1** (monovalent ligand conjugated to the extended rod). As shown by the Cheng-Prussof equation, the IC_50_ value of a competitive inhibitor is always higher than its *K*_D_.^34^ Thus, the affinity is often underestimated with the IC_50_ suggesting that real affinity difference between **1** and **3.1** is probably lower than the factor of 6; however it also suggests that the rod scaffold slightly contributes to the binding affinity. For this reason, compound **3.1** will be used as monovalent ligand of reference to calculate the valency-corrected β-factors. Indeed, a β-factor of 8 is calculated for the divalent ligand **3.2** (*K*_Dapp_ = 2.4 μM), suggesting that a chelation mechanism is now operative. The binding responses of **3.2** compared to its monovalent counterpart **3.1** (see Figure S1 and S2) highlight the multivalent binding properties of **3.2**. Figure S2 shows that while **3.1** displays a hyperbolic 1:1 monovalent binding curve, **3.2** binding goes through a multivalent binding mode, chelation, and/or clustering, in the μM range of concentration before it reaches a *R*_max_ of half the value of **3.1**. The avidity is further boosted by increasing the ligand valency to six using flexible linkers in **3.6** totaling to a valency-corrected β-factor of 570 (Table 1). This occurs despite the entropy loss caused by the presence of a flexible linker in **3.6**, which we can estimate contributes by a negative factor of 2, as judged by comparison of the inhibition data for **3.2** and the **3.2-long** control obtained in ref (14) (see Table 1, entries 3 and 4). The affinity improvement from **3.2** to **3.6** (2 orders of magnitude) reveals the positive effect of increasing the local concentration of ligand **1** at each extremity of the rigid spacer, due to statistical rebinding effects promoted locally at the level of each extremity with the corresponding DC-SIGN binding sites (note that chelation is not permitted, for distance reasons, by the 3 spearheads on each extremity of **3.6**). Thus, the cumulative effects of chelating/clustering and statistical rebinding modes are responsible for the high binding potency of **3.6**.

To further analyze the interaction mode of compound **3.6**, complementary biophysical solution-based approaches were used. Using the intrinsic fluorescence properties of the ROD spacer (λ_emission_ = 430 nm/λ_excitation_ = 390 nm), a DC-SIGN ECD tetramer titration assay (Figure 2C), by fluorescence polarization (FP), confirms the nM range affinity (EC_50_ = 68 ± 8 nM). Interestingly, a plateau is reached for a **3.6**/DC-SIGN ECD stoichiometry around 1:1 (400 μM of both partner) suggesting an avidity mainly based on a chelation binding mode. A closer examination of the **3.6**/DC-SIGN tetramer binding process was performed by sedimentation velocity measurement at 4 μM of the DC-SIGN tetramer alone and with increasing concentrations of dendrimer (from 5 nM to 6 μM range) (Figure 2D). The present experiment revealed a first single species with an *s*_20,w_ = 4.7 S with a molar mass of 149 kDa. The peak is compatible with an elongated DC-SIGN tetramer. The same peak is observed for the DC-SIGN ECD solution alone and with concentrations of compound **3.6** going from 5 nM to 500 nM. For higher concentrations (μM) of the compound, a second species appeared at an *s*_20,w_ = 7.3 S, corresponding to a dimer of DC-SIGN tetramers. The experiment shows that, as the ligand concentration reaches the μM range and approaches the protein concentration, clustering becomes a relevant binding mode, driven by the decrease of available DC-SIGN binding sites belonging to the same tetramer and suggesting an alternative mechanism of avidity generation in different concentration ranges of **3.6**.

**Figure 3:**
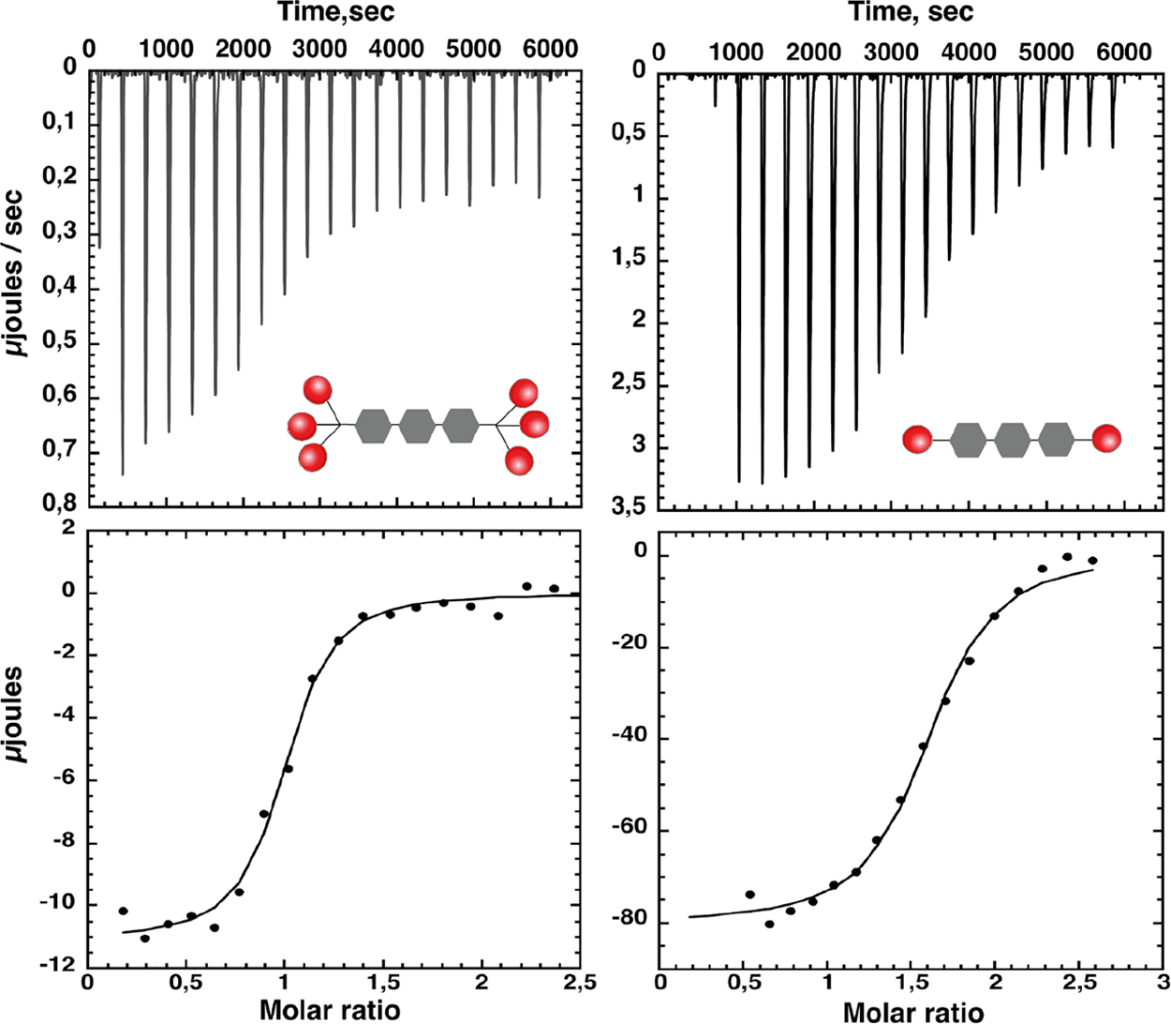
Isothermal titration microcalorimetry of compound **3.2** and **3.6**. Titration calorimetry of compound **3.6** (30 μM, left panel) and compound **3.2** (375 μM, right panel) in a cell containing DC-SIGN tetramer (respectively 30 μM and 48.75 μM).

Thermodynamic parameters of multivalent binding as measured by isothermal titration calorimetry (ITC) were analyzed for compounds **3.2** and **3.6**. Usually, ITC should provide, among other parameters, a “real” *K*_D_ value; however, here we prefer to refer to a *K*_Dapp_. Indeed, in these ITC experiments, we are not evaluating an affinity (a 1:1 unitary interaction, defined by *K*_D_) but rather an avidity phenomenon resulting from cumulative unitary bonds (each of whose individual contributions to the overall avidity cannot be deconvoluted). In addition, this avidity results from several multivalent modalities, a population of binding modes. Thus, the use of *K*_Dapp_, instead of *K*_D_, emphasizes that the values determined here are the result of a complex phenomenon. The ITC binding analysis (Figure 3 and Table 2) is fully in agreement with the other biophysical techniques (see Table 2 for SPR and Figure 2C for FP of **3.6**) with *K*_Dapp_ values equal to 58 ± 1 nM and 1.1 ± 0.2 μM for compounds **3.6** and **3.2**, respectively. The enthalpy-driven association is significantly improved for compound **3.6** compared to **3.2** due to displaying of six copies of **1**, rather than two, which drives the equilibrium toward the bound states. Increasing the local concentration close to the site is a major source of avidity generation. The Δ*H* difference is offset by a more unfavorable entropy for **3.6**, which probably originates from the loss of degrees of freedom of construct extremities, six versus two, upon binding. Hydrophobic effect may also contribute, since the hydrophobic rod is shielded better from the solvent by the six units of ligand **1** in **3.6**(35) and exposing the hydrophobic parts and ordering the solvent around them is entropically costly. Combining the enthalpy and entropy effects, the Δ*G* for the hexavalent ligand improves by 10 kJ/mol. It is interesting to note that a stoichiometry of 1 for the complex with **3.6** is observed, supporting again the chelation mode. On the contrary, a stoichiometry *n* = 1.5 is observed for **3.2**, with a *K*_Dapp_ in the μM range, which is compatible with a mixed chelation and clustering binding mode. Again, clustering is observed here in the μM range, as observed for **3.6** by analytical ultracentrifugation (Figure 2D), suggesting that this binding mode, at least in solution, appears at a specific relative ratio of concentration between DC-SIGN and the ligands.

**Table 2:** Thermodynamic Parameters of Multivalent Binding between Compounds **3.2** and **3.6** toward DC-SIGN as Measured by ITC

Compounds	*K*_Dapp_(nM)	Δ*G*, kJ/mol	Δ*H*, kJ/mol	*T*Δ*S*, kJ/mol	*n*
**3.2**	1100 ± 200	–30.87	–85.27 ± 4.48	–54.4	1.56 ± 0.04
**3.6**	58 ± 1	–41.28	–146.98 ± 6.03	–105.7	0.96 ± 0.03

Thus, the data discussed so far support the view that the length of the rod, the presence of flexible linkers, and the overall valency of the compounds influence the affinity values. For a rod of optimal length, the affinity increases by 1 order of magnitude when chelation becomes attainable (**3.1** vs **3.2**) and 2 orders of magnitude when statistical rebinding effects become operative on top of chelation (**3.2** vs **3.6**). However, the affinity dependence on the rod size in the series **1.6**, **2.6**, **3.6** and its brusque increase by 2 orders of magnitude when reaching **3.6** demand further explanation. Additionally, the sedimentation velocity experiments suggest that protein clustering may become a significant binding mode in the appropriate relative concentration range of ligand and lectin. Geometrical modeling suggests that the spearhead distance in **3.6** can bridge additional CRDs from other tetramers, as compared to **1.6**. and **2.6,** when densities of spearheads and binding site allow it (Figure S4).

**Figure 4:**
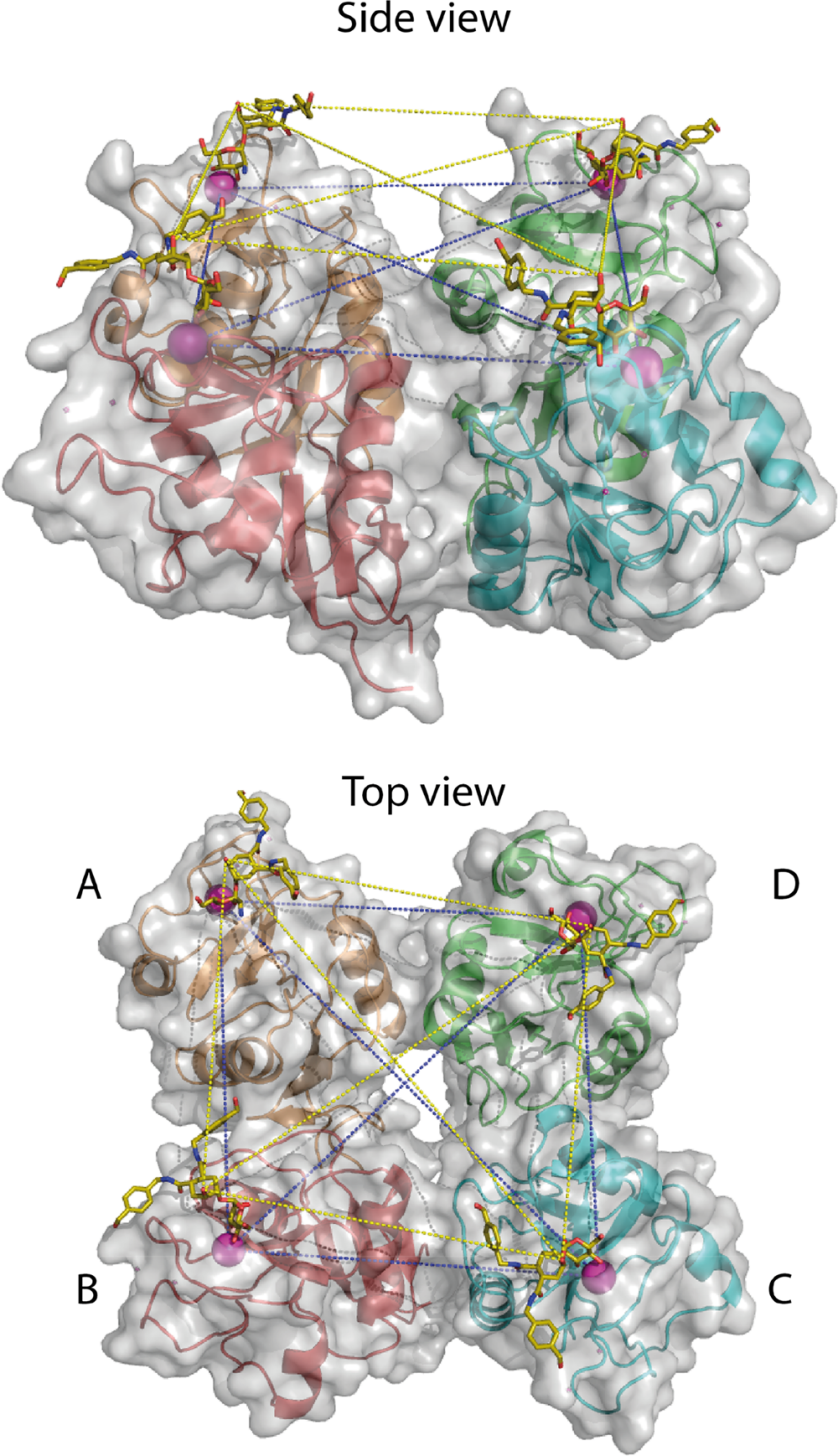
Distances between binding sites within tetrameric head of DC-SIGN. Tetrameric model with compound **1** in each CRDs was derived from combination of SAXS envelope of DC-SIGN ECD,^9^ PDB: 6GHV,^15^ for glycomimetic/CRD complex and STD-NMR data for **1**.^12^ Distances between Ca^2+^···Ca^2+^ and OBG···OBG (OBG is the anomeric oxygen atom of **1**) are represented by dark blue or yellow lines, respectively. In the top view, CRDs are identified by letters from A to D (corresponding to orange, red, cyan and green cartoons respectively), allowing reference to the corresponding distances in Table 3 for this static model. For clarity, only the Ca^2+^ atoms of the carbohydrate binding sites are shown (magenta).

**Figure 5:**
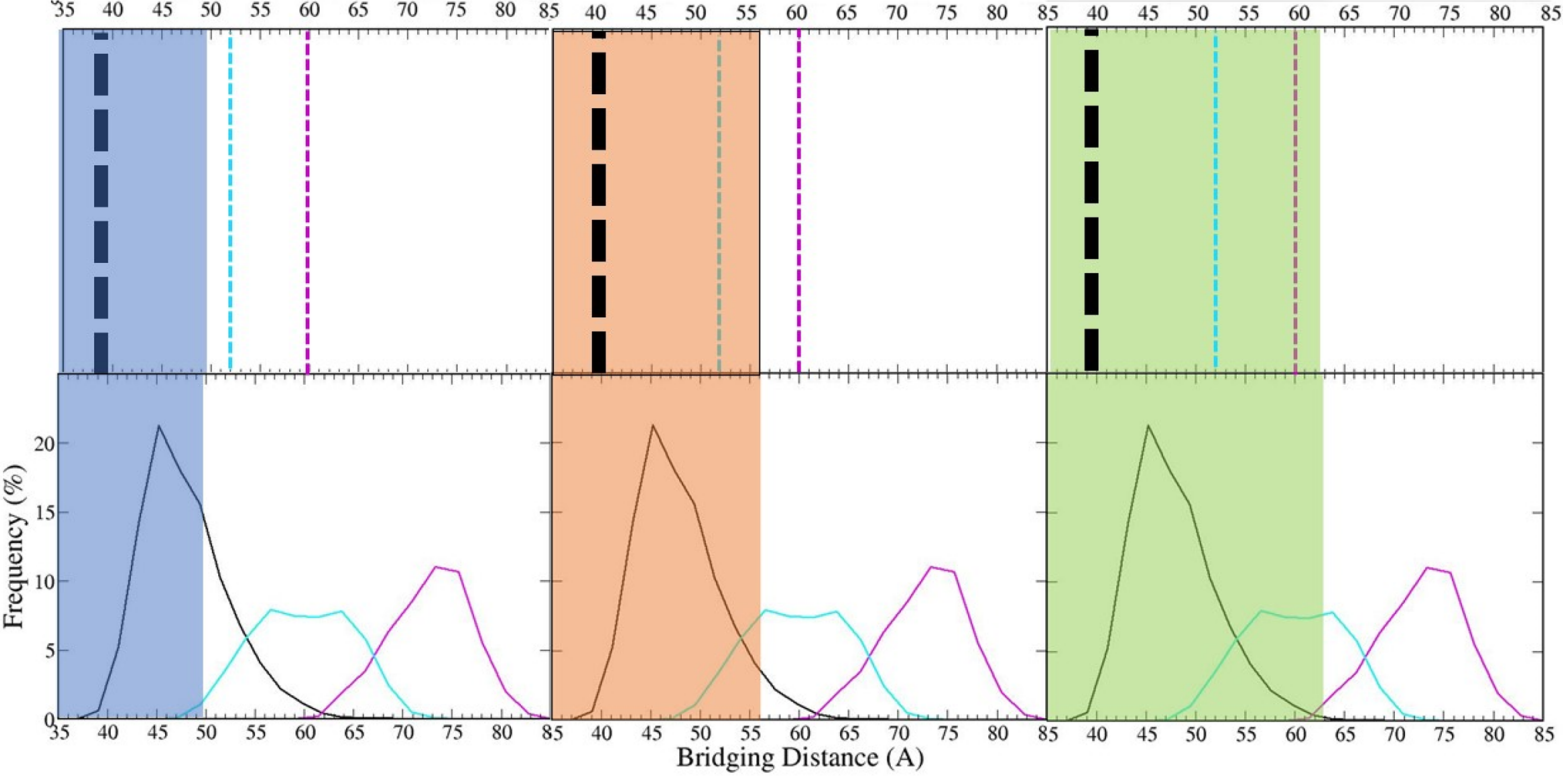
Bridging Ca^2+^···Ca^2+^ distances in DC-SIGN tetramer. Static (dashed lines, top) and populations of dynamic (solid curves, bottom) distances are colored black for *Side* (left), cyan for *Diag 1* (middle), and magenta for *Diag2* (right). The chelating potentials of compounds **1.6**, **2.6**, and **3.6** are shown as colored boxes (blue, orange, and green, respectively).

**Figure 6:**
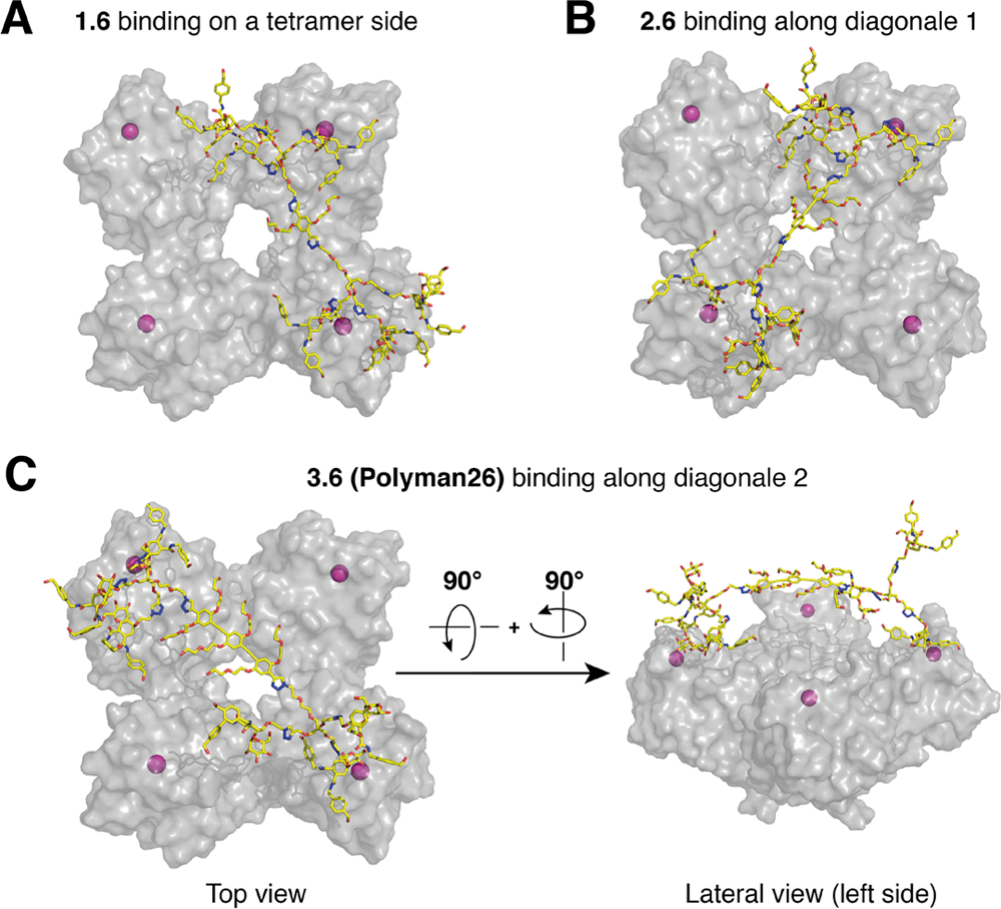
Computational models for a selection of chelation binding modes of rod-based dendrimers (in CPK colored sticks) on tetrameric DC-SIGN head. A. Chelation binding of **1.6** on the *Side*. B. Chelation binding of **2.6** along *Diag1.* C. Chelation binding of **3.6** (Polyman 26) along *Diag2.* Tetrameric head of DC-SIGN is represented in a surface mode and in the same orientation as in Figure 4. For clarity, only the Ca^2+^ atoms of the carbohydrate binding sites are represented (magenta).

To gain structural insight into the possibilities of multivalent binding, we modeled the chelating binding modes attainable by compounds **1.6**, **2.6**, and **3.6** in the DC-SIGN tetramer. In the static model,^9^ we distinguish three types of Ca^2+^···Ca^2+^ distances in the DC-SIGN CRDs to be bridged by the compounds (Figure 1B; Table 3 and Figure 4): a *Side* distance around 40 Å and two different diagonal distances *Diag1* (52 Å) and Diag2 (60 Å). To estimate direct through-space linking, we considered the OBG···OBG distances of **1** bound to the DC-SIGN CRDs (at most 4.1 Å shorter than the respective Ca^2+^···Ca^2+^ distances; Table 3) coupled with the maximal OBG···OBG distances of unbound **1.6**, **2.6**, and **3.6** compounds (45.5, 52.0, and 58.6 Å, respectively) obtained from high-temperature dynamics in implicit solvent. We thus get the chelating potential (i.e., maximal Ca^2+^···Ca^2+^ distance which can be bridged) of 49.6, 56.1, and 62.7 Å, respectively (Figure 5). This would mean that **1.6** would only be capable to bind in the *Side* mode, **2.6** would add *Diag1*, and **3.6** could use all three chelation binding modes (Figure 5 top; Figure 6).

**Table 3:** Ca^2+^···Ca^2+^ and OBG···OBG Distances Measured in the Model of the DC-SIGN Tetramer/**1** Complex (Å)^a^

Distance (Å)	Ca^2+^···Ca^2+^ (static)	OBG···OBG	difference	Ca^2+^···Ca^2+^ (dynamic)
Side				
AB	40.3	36.7	3.6	46.9
AD	40.3	39.9	0.4	
BC	38.7	37.1	1.6	
CD	39.4	35.3	4.1	
Diag				
BD (Diag 1)	52.1	48.3	3.8	58.7
AC (Diag 2)	59.9	56.8	3.1	71.6

a OBG is the anomeric oxygen atom of **1** (EZ8 residue in compound 16 from ref (15)).

**Figure 7:**
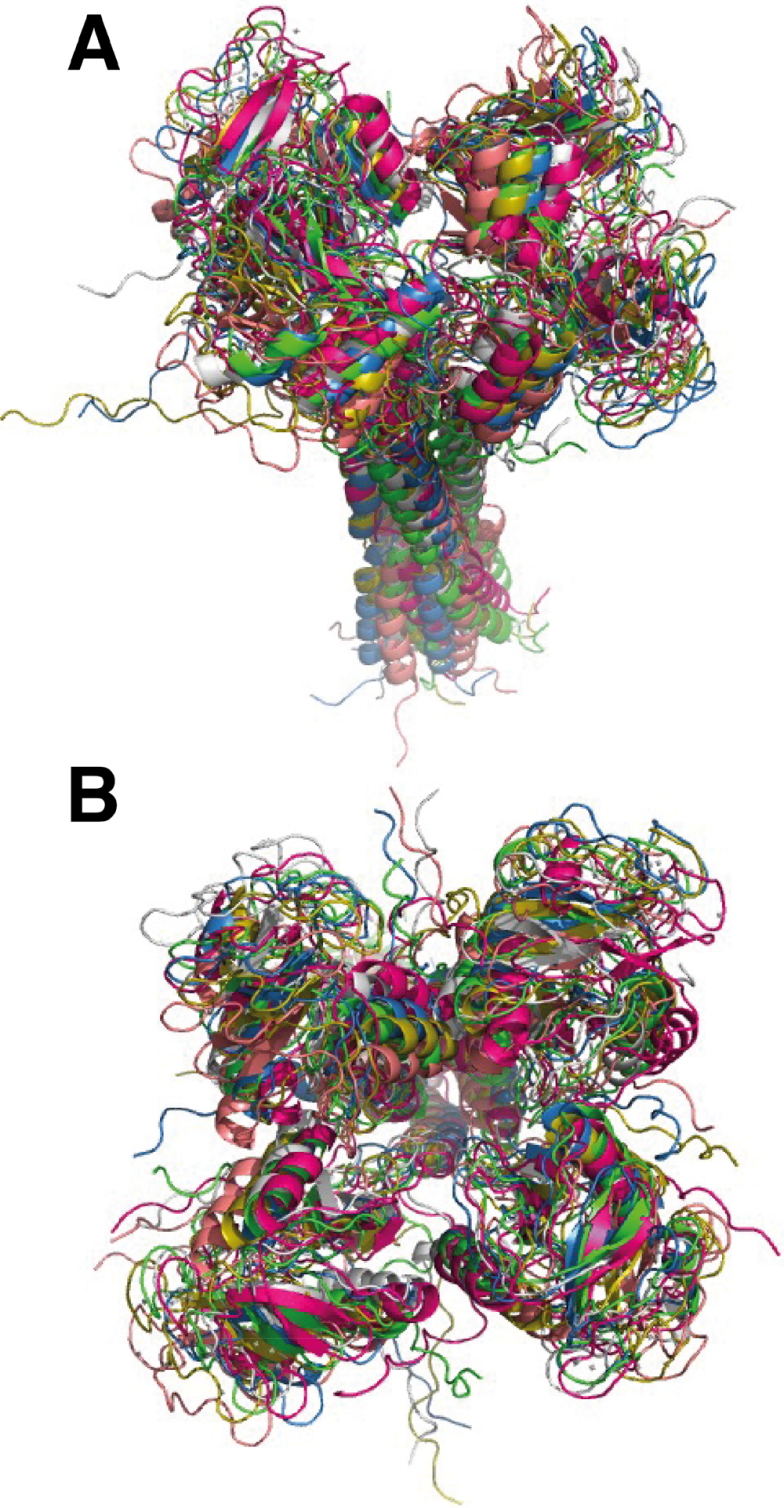
Snapshots from the 2 μs MD of DC-SIGN tetramer A. Side view, B. top view. For better visualization of DC-SIGN dynamics, see Movies S1 and S2.

A more realistic picture arises from a dynamic description of the DC-SIGN tetramer. Contrary to many other multivalent lectins, whose carbohydrate binding sites have a fixed relative geometry, movements of the four CRDs within the tetrameric DC-SIGN are possible and have been demonstrated previously.^9,36^ They are enabled by the flexible link connecting the CRDs to the neck. It is thought that such flexibility is at the root of DC-SIGN capabilities to recognize and adapt to a wide repertoire of pathogen glycans whereas other lectin receptors, with fixed site spacing, recognize limited molecular patterns. Strikingly, this flexibility, and thus inter-CRDs distances variability, is rarely taken into consideration within the plethora of work aiming at designing multivalent ligand targeting DC-SIGN.^37−41^ In order to evaluate its impacts on the observed avidity boost, the conformations attained during 2 μs of explicit-solvent molecular dynamics (MD) simulation of the DC-SIGN tetramer/neck model are shown in Figure 7 (see also in Supporting Information Movies S1 and S2).

The average CRD separations (measured as the Ca^2+^···Ca^2+^ distances) from MD are by 7–12 Å larger than those in the average low-resolution SAXS model (Table 3). Considering less populated DC-SIGN tetramer conformations, the difference can increase up to 22–26 Å (Figure 5 bottom; solid curves). Thus, both **1.6** and **2.6** would capture a large portion of the *Side* binding modes (78% and 97%, respectively). Compound **2.6** would add 19% of *Diag1* modes. The longer **3.6** can capture not only all the *Side* binding modes but also 37% of *Diag1* binding modes, and it also starts to capture the shortest dynamic *Diag2* binding modes even if most of them seem to be mostly beyond its reach (Figures 5 and 6).

While all data point toward a combination of statistical binding and chelation, improved by CRDs flexibility, as a source of the nM avidity range for the compound **3.6**, still, a protein clustering effect also remains a possibility. Such a clustering mode has been observed here in the μM range of affinity, in AUC experiments, where DC-SIGN ECD was in solution. Such clustering can also occur on the surface of cell membranes, due to the lateral mobility of the embedded tetramers and is used by glycosylated enveloped viruses whose particle size can bridge easily several receptors on the surface. It requires proximity and thus high density of DC-SIGN receptors. The similar avidity values obtained comparing results in solution from fluorescence polarization and ITC on one side and direct interaction with oriented surfaces in SPR on the other side (where clustering mode could have been favored) argues here for a marginal effect of clustering in **3.6** avidity, at least in the conditions tested here. We have elaborated some theoretical thoughts regarding the potential clustering abilities of the various rod-based dendrimers toward DC-SIGN-surfaces (Section 7 in Supporting Information). These considerations suggest that for these rod-dendrimers, which can span a distance far smaller than a virus particle, clustering events will not significantly account for the avidity properties toward lectins receptors in this context.

Recently, we have shown that DC-SIGN and related L-SIGN are attachment factors of SARS-CoV-2 virus and contribute to the infection.^7^ Many other studies have followed, confirming these observations and even suggesting that these lectin receptors could be the primary receptors on their own and also contribute to immune dysregulation during SARS-CoV-2 infection.^42−45^ In that context, we also demonstrated that Polyman26 (compound **3.6** in this work) could be used in cellular assay to inhibit DC-SIGN dependent trans-infection of SAR-CoV-2 virus.^7^

The rigorous deciphering of the binding of **3.6** should help to improve the design of multivalent compounds and to predict the avidity generation of promising antagonists of DC-SIGN and, more broadly, of other oligomeric receptors. In the context of the SARS-CoV-2 infection, L-SIGN could be a next target of interest since it is present at the surface of ACE2+ endothelial cell and is suggested to play a role as a coreceptor.^42^ Next generation of improvement can be based on higher affinity monovalent ligands, higher valency, but also scaffold geometry. We have already started to develop mimetic targeting L-SIGN and not only DC-SIGN.^46^ However, aside to the ligand selectivity, the topological aspects underlined here are an important source of explanation for the avidity differences between multivalent ligands. Recent work, using glyconanoparticles as binding probes, has shown the drastic difference of multivalent binding mechanisms between the two closely related tetrameric DC-SIGN and L-SIGN receptors. While DC-SIGN shows a propensity to chelation binding mode, L-SIGN appears to generate inter-cross-linking binding modes (equivalent of clustering but in solution) toward the glyconanoparticles.^41^ Thus, despite a closely conserved sequence between the two tetrameric lectins (77%), major structural differences in the CRD head presentations are involved in multivalency. This is in line with previous structural work attempting to define respective organization of DC-SIGN and L-SIGN tetramers in which the carbohydrate binding sites are presented on top of the tetramer (DC-SIGN) or laterally (L-SIGN).^9,47^ Thus, targeting capacity toward L-SIGN may require not only new specific ligands but also an appropriate multivalent scaffold able to match to the specific topology of its CRDs. As for DC-SIGN, flexibility of CRDs within L-SIGN tetramers have been documented.^48^ The strategy presented here to address the avidity toward DC-SIGN could be used to develop new virus-attachment blockers in the future for L-SIGN and other C-type lectin receptors of interest.

## Conclusion

Combining a range of biophysical techniques (partners in solution or immobilized) and molecular modeling, we have deciphered different binding modes and sources of avidity of glycomimetics with controllable rigid spacer length and multivalent presentation toward multimeric lectin receptors. In this case study, the cumulative chelating and statistical rebinding modes make 2 orders of magnitude difference for the compound **3.6** to become a potent antagonist. It is important to note that this avidity level, a *K*_Dapp_ around 10 nM, is reached here with only a hexavalent presentation, as compared to other optimized multivalent glycoclusters targeting DC-SIGN tetramer which need to present up to 16 ligands to reach similar avidity.^20,21^ The molecular reason behind this efficiency was a long rigid core to cover part of the distance between adjacent sites and flexible extremities with trivalent presentation to facilitate adjustment and statistical rebinding at each individual binding site level. Moreover, this extended rigid core favors sufficient extension of the structure in solution to potentiate diverse chelation binding modes. Indeed, when considering protein dynamics, it enabled to cover *Side* and *Diag1* dynamic distances and even marginally some *Diag2* chelation binding mode. This unique combination of chelation and statistical-rebinding with the capacity to adapt to the structural plasticity of the receptor is the secret of the net boost in avidity.

## Supporting Information Available

Materials and methods. Synthesis of monovalent compounds control dendrimer 3.1, SPR direct interaction, oriented surface functionalization, isothermal calorimetry analysis, fluorescence polarization assay, analytical ultracentrifugation analysis, molecular modeling, and considerations on clustering binding mode (PDF)
Movie S1: DC-SIGN dynamics (MPG)
Movie S2: DC-SIGN dynamics (MPG)
Transparent Peer Review report available (PDF)

## Supplementary Material

oc2c01136_si_001.pdf

oc2c01136_si_002.mpg

oc2c01136_si_003.mpg
