# Supplementary material for: Powerful Avidity with a Limited Valency for Virus-Attachment Blockers on DC-SIGN: Combining Chelation and Statistical Rebinding with Structural Plasticity of the Receptor
Source: ACS Cent Sci. 2023 Feb 20;9(4):709–18. doi: 10.1021/acscentsci.2c01136 (PMC10141607; doi:10.1021/acscentsci.2c01136)
Supplement: Supplementary file 1 — oc2c01136_si_001.pdf [file oc2c01136_si_001.pdf]

Supporting information :

Powerful avidity with a limited valency for virus-attachment blockers on DC-SIGN: Combining chelation and statistical binding with structural plasticity of the receptor

Vanessa Porkolab,[†,a] Martin Lepšík[¶,x,a], Stefania Ordanini, [§] Alexander St John,[∂] Aline Le Roy, [†] Michel Thépaut,[†] Emanuele Paci,[¥] Christine Ebel, [†] Anna Bernardi,[§,*] Franck Fieschi[†,*]

[†] Univ. Grenoble Alpes, CNRS, CEA, Institut de Biologie Structurale, 38000 Grenoble, France. Email: franck.fieschi@ibs.fr
[¶] Univ. Grenoble Alpes, CNRS, CERMAV, 38000 Grenoble, France
[x] Institute of Organic Chemistry and Biochemistry, Czech Academy of Sciences, Flemingovo nam. 2, Prague 6, 166 10, Czechia
[§] Universita` degli Studi di Milano, Dipartimento di Chimica, via Golgi 19, 20133, Milano, Italy.
[∂] Astbury Centre & School of Molecular and Cellular Biology, University of Leeds, Leeds, UK
[¥] Department of Physics and Astronomy "Augusto Righi", University of Bologna, Bologna, Italy

[a] Both authors contributed equally

[*] Corresponding authors:

email: anna.bernardi@unimi.it

email: franck.fieschi@ibs.fr

1. Synthesis 2
  1.1. Synthesis of monovalent control glycodendrimer 3.1 3
2. SPR direct interaction 6
  2.1. DC-SIGN S-ECD surface functionalization 6
  2.2. Compound titration over a DC-SIGN S-ECD surface 6
3. Isothermal titration calorimetric analysis 8
4. Fluorescence polarization (FP) assay 9
5. Analytical Ultracentrifugation analysis 9
6. Molecular Modeling 9
  6.1. Compound building and parametrization. 9
  6.2. High-temperature MD of compounds in implicit solvent 10
  6.3. Binding Modes of Compounds to Static DC-SIGN Tetramer 10
  6.4. Modeling DC-SIGN Tetramer Including Neck 11
  6.5. Dynamics of DC-SIGN Tetramer Including Neck 11
7. Considerations on clustering binding modes 12

1. Synthesis

The synthesis of compound **1** is described in Varga et al [1].

With the exception of the monovalent control **3.1** which is described below, the synthesis and characterization of all the other dendrimers is reported in ref [2]. For sake of clarity, the following table correlates the compound numbering in this and previous papers.

Table S1. Compound numbering and structures in this work and refs [2], [3].

This paper	Ref [2]	Ref [4]	Compounds	Symbol
**3.1**			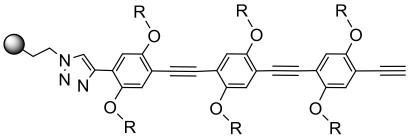	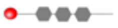
**3.2**	**3.4**		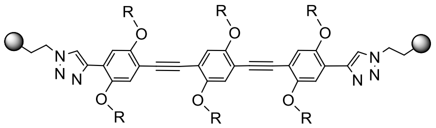	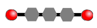
**3.2-long**	**3.7.6**		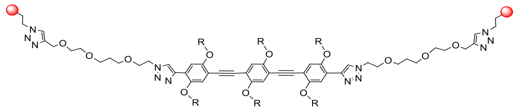	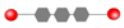
**1.6**	**1.5.4**		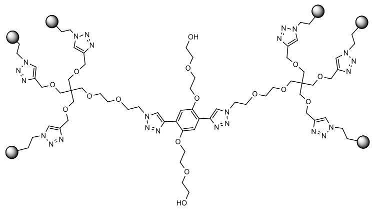	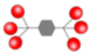
**2.6**	**2.5.4**		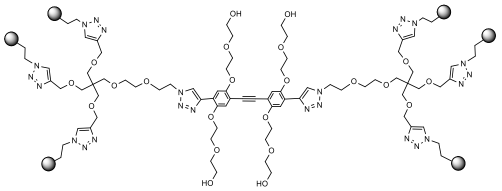	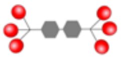
**3.6**	**3.5.4**	**PM26**	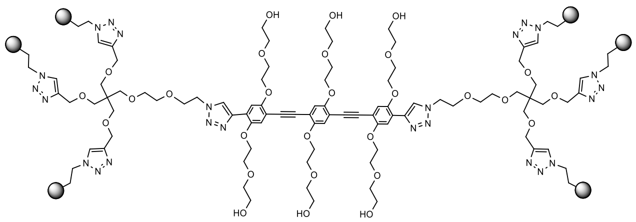	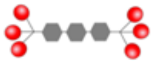

1.1. Synthesis of monovalent control glycodendrimer 3.1

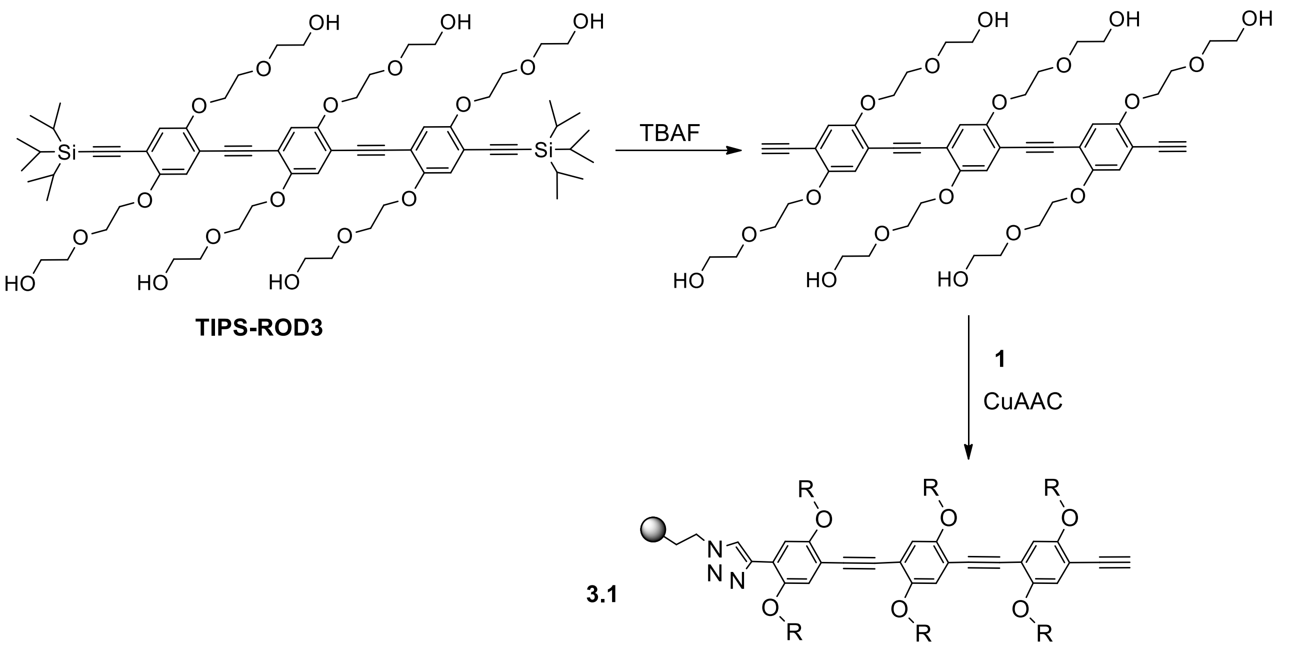

Scheme S1.

To a stirred solution of **TIPS-ROD3** [1] (6.4 mg, 5.0 μmol, 1 eq.) in THF (200 μL), TBAF (1 M in THF, 10.0 μL, 2 eq.) was added under nitrogen. After 1 h, TLC analysis (CH_2Cl_2 : MeOH 9:1) showed that the desilylation reaction was complete. The CuAAC reagents were added as solutions in the following order: TBTA (Tris ((1-benzyl-4-triazolyl) methyl) amine) in THF (0.53 mg, 1.0 μmol, 0.2 eq., 100 μL of THF), $CuSO_4{\cdot}5H_2O$ in H_2O (0.12 mg, 0.5 μmol, 0.1 eq., 80 μL of H_2O) and sodium ascorbate in H_2O (0.40 mg, 2.0 μmol, 0.4 eq., 80 μL of H_2O). The reaction mixture was stirred at room temperature, under nitrogen atmosphere and in the dark for 10 minutes. Finally ligand **1**[1] (2.7 mg, 4.0 μmol, 0.8 eq.) was added as solid. THF and H_2O volumes were adjusted to 250 μL each. The reaction was stirred at RT, under nitrogen and in dark overnight. TLC analysis (CH_2Cl_2 : MeOH 9:1) revealed total consumption of the ROD; TLC (CH_2Cl_2:MeOH:H_2O 75:25:2.5) monitored the formation of a new polar species, which MALDI MS analysis (DHB matrix) confirmed to be the expected monovalent product, together with small amounts of divalent **3.2** and residual starting material. Quadrasil™-MP (S/Cu 15:1, 5 mg) was added to the reaction mixture, which was stirred for 10 minutes, and then filtered. The solvent was removed under reduced pressure from the filtrate and the resulting mixture was redissolved in MeOH and purified by size exclusion chromatography (Sephadex LH-20, MeOH, 1 drop in 4 seconds), obtaining 2.3 mg of **3.1** (yield 28 %).

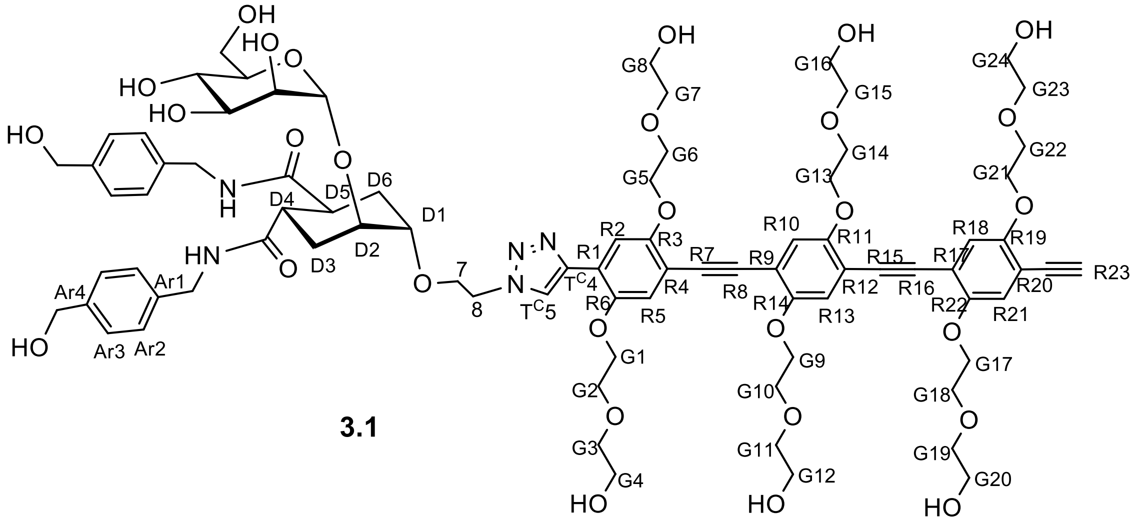

3.1

Scheme S2.

^{1}H NMR (400 MHz, CD_3OD): δ 8.63 (s, 1H, H_{TC5}), 7.85 (s, 1H, H_{R2}), 7.31 – 7.09 (m, 12H, H_{Ar2}, H_{Ar3}, H_{R5}, H_{R10}, H_{R13}, H_{R18}, H_{R21}), 4.71 – 4.64 (m, H_8), 4.59 – 4.53 (m, 4H, C$\underline{H_2}$OH), 4.35 – 4.13 (m, 16H, C$\underline{H}_2$NH, H_{G1}, H_{G5}, H_{G9}, H_{G13}, H_{G17}, H_{G21}), 4.07 – 3.98 (m, 2H, H_7), 3.97 – 3.80 (m, 16H, H_{D2}, H_2, $H_{G2,}$ H_{G6}, H_{G10}, H_{G12}, H_{G14}, H_{G18}, H_{G22}), 3.79 – 3.60 (m, 26H, H_{6a}, H_{6b}, H_3, H_{D1}, H_{G3}, H_{G7}, H_{G11}, H_{G15}, H_{G19}, H_{G23}, H_{G4}, H_{G8}, H_{G16}, H_{G20}, H_{G24}), 3.60 – 3.46 (m, 2H, H_5, H_4), 3.59 – 3.45 (m, 3H), 2.90 – 2.73 (m, 2H, H_{D4}, H_{D5}), 1.96 – 1.72 (m, 6H, H_{D3}, H_{D6}).

^{13}C NMR (100 MHz, CD_3OD): δ 177.01, 176.60 ($\underline{C}$ONH), 155.65, 155.45, 155.08, 154.97, 154.81 (C_{R6}, C_{R11} C_{R14}, C_{R19}, C_{R22}), 150.72 (C_{R3}), 143.74 (C_{TC4}), 141.59, 141.53, 139.14, 138.92 (C_{Ar1}, C_{Ar4}), 128.38, 128.31, 128.19, 128.15 (C_{Ar2}, C_{Ar3}), 127.26 (C_{TC5}), 122.43 (C_{R1}), 119.86, 119.22, 119.04, 118.64 (C_{R5}, C_{R10}, C_{R13}, C_{R18}, C_{R21}), 116.23, 116.16, 115.67, 114.90, 114.44 (C_{R4}, C_{R9}, C_{R12}, C_{R17}, C_{R20}), 112.31 (C_{R2}), 100.31 (C_1), 76.01 (C_5), 75.55 (C_3), 74.10 (C_{G3}, C_{G7}, C_{G11}, C_{G15}, C_{G19}, C_{G23}), 72.54 (C_2), 72.36 (C_{D2}), 72.15 (C_{D1}), 71.26 – 70.55 ($C_{G2,}$ C_{G6}, C_{G10}, C_{G14}, C_{G18}, C_{G22}, C_{G1}, C_{G5}, C_{G9}, C_{G13}, C_{G17}, C_{G21}), 69.22 (C_7), 69.03 (C_4), 64.88 ($\underline{C}H_2$OH), 63.01 (C_{G12}), 62.55 – 62.16 (C_6, C_{G4}, C_{G8}, C_{G12}, C_{G16}, C_{G20}, C_{G24}), 51.45 (C_{G8}), 43.67 ($\underline{C}H_2$NH), 41.95 (C_{D4}, C_{D5}), 25.56 (C_{D3}, C_{D6}).

MS (ESI-HRMS): *m/z* calculated for $[C_{82}H_{105}N_5O_{29}Na_2]^{2+}$: 834.8340; found: 834.8357.

$[\alpha]_D{}^{25}$: -16.4 (c = 0.1, MeOH).

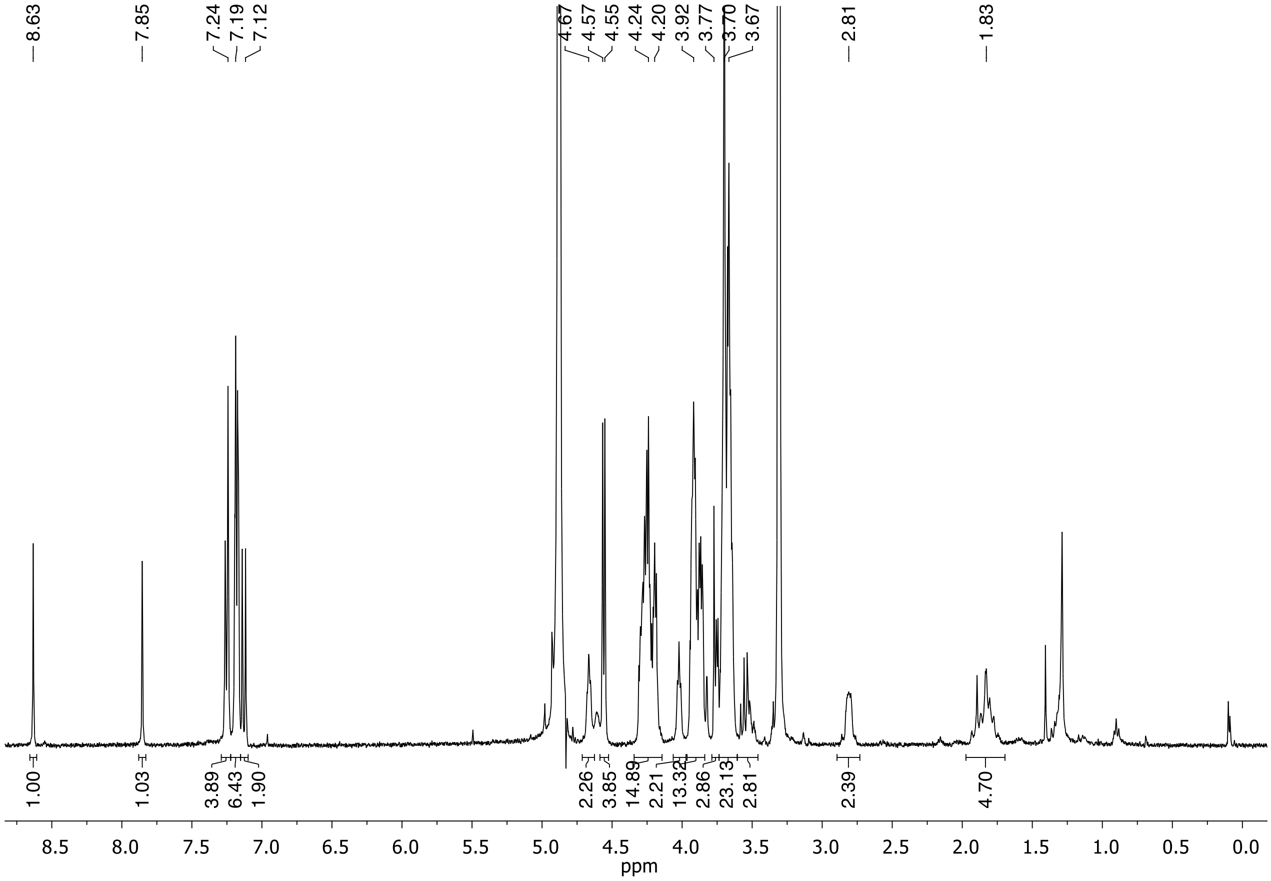
8.63
7.85
7.24
7.19
7.12
4.67
4.57
4.55
4.24
4.20
3.92
3.77
3.70
3.67
2.81
1.83
1.00
1.03
3.89
6.43
1.90
2.26
3.85
14.89
2.21
13.32
2.86
23.13
2.81
2.39
4.70
ppm

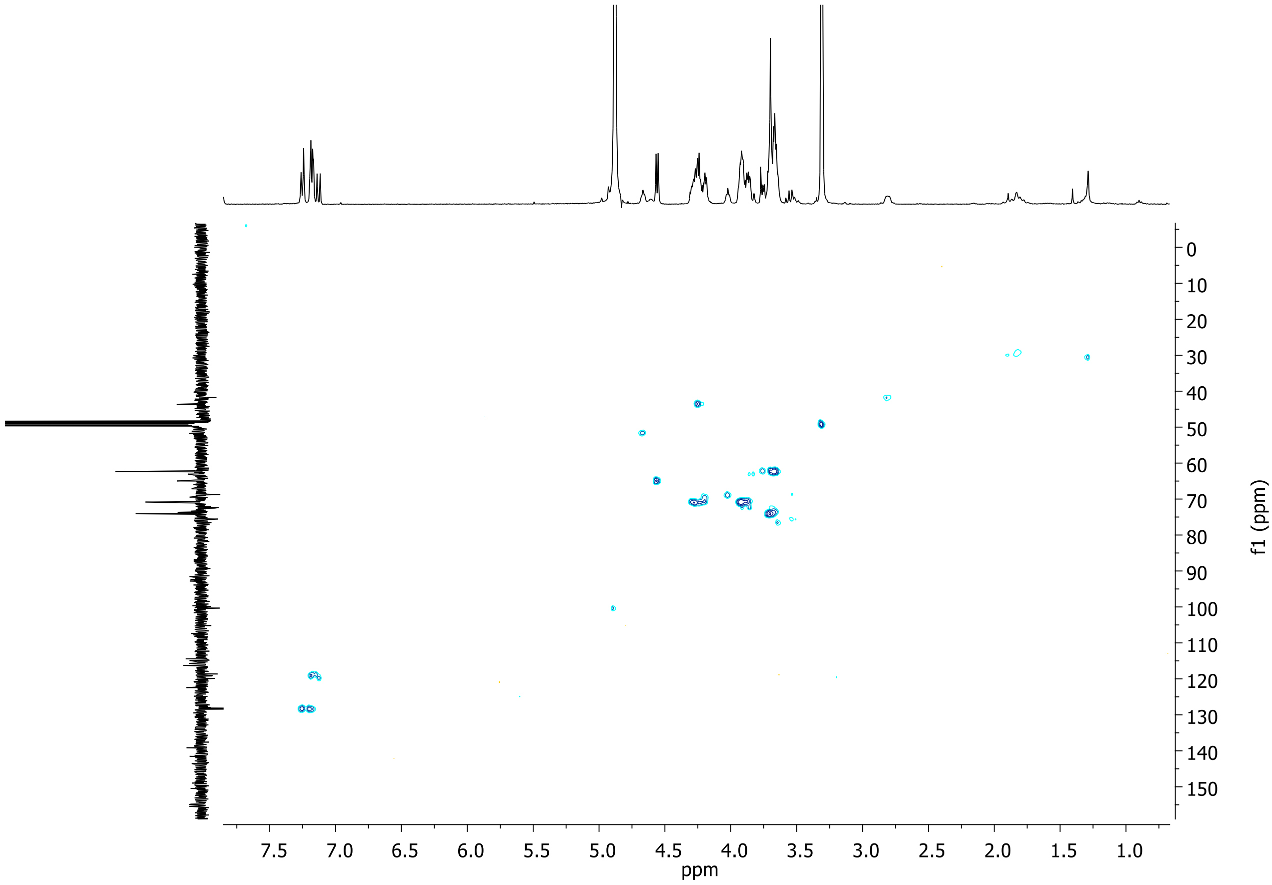
ppm
f1 (ppm)

2. SPR direct interaction

2.1. DC-SIGN S-ECD surface functionalization

DC-SIGN S-ECD was overexpressed and purified as described in Porkolab *et al.*[5]. SPR experiments were performed on a Biacore T200 using a CM3 chip, functionalized at 5 μL/min. *Strep*Tactin (IBA company) following by DC-SIGN S-ECD were immobilized on flow cells using amine-coupling method.
Flow cell (Fc) 1 was prepared as reference surface. Flow cell (Fc) 1 to 4 were activated with 50 μL of a 0.2M EDC/ 0.05 M NHS mixture. After this step, Fc1, Fc2, Fc3 and Fc4 were functionalized with 170 µg/mL *Strep*Tactin, and then remaining activated groups of all cells were blocked with 80 μL of 1 M ethanolamine. After blocking, the four Fc were treated with 5 μL of 10 mM HCl to remove no-specific bound protein and 5 μL of 50 mM NaOH/ 1M NaCl to expose surface to regeneration protocol. Finally, an average of 2300 RU of *Strep*Tactin was immobilized on each surface. This previous procedure was repeated for the functionalization of DC-SIGN ECD with its injection at 49.7 µg/mL. The final RU obtained after the washing steps are 2278 RU on Fc2, 2316 RU on Fc3, 2300 RU) on Fc4.

2.2. Compound titration over a DC-SIGN S-ECD surface

For direct interaction studies, increasing concentrations of compound were prepared in a running buffer composed of 25 mM Tris pH 8, 150 mM NaCl, 4 mM $CaCl_2$, 4%DMSO, 0.05% P20 surfactant, and 85 µL of each sample was injected onto the surfaces at 30 µL/min flow rate. The resulting sensorgrams were reference surface corrected.
The apparent affinity of compounds was determined by fitting the *steady state affinity model* (Eq. 1) to the plots of binding responses versus concentration.

$$R_{eq} = \frac{K_A \cdot C \cdot R_{\max}}{1 + K_A} \qquad \text{Eq. 1}$$

R_{eq} is equilibrium binding response, K_A equilibrium association constant ($K_D = 1/K_A$), C concentration of the injected analyte, R_{max} surface binding capacity.
K_d obtained reflects the affinity for the surface and not for individual lectin receptor. However, this mode of multisite interaction onto a surface is closer to the real interaction mode at the cell surface and its stoichiometric interaction with a lectin receptor is considered as 1:1 by avidity generation.

Compound **3.1**	Compound **3.2**

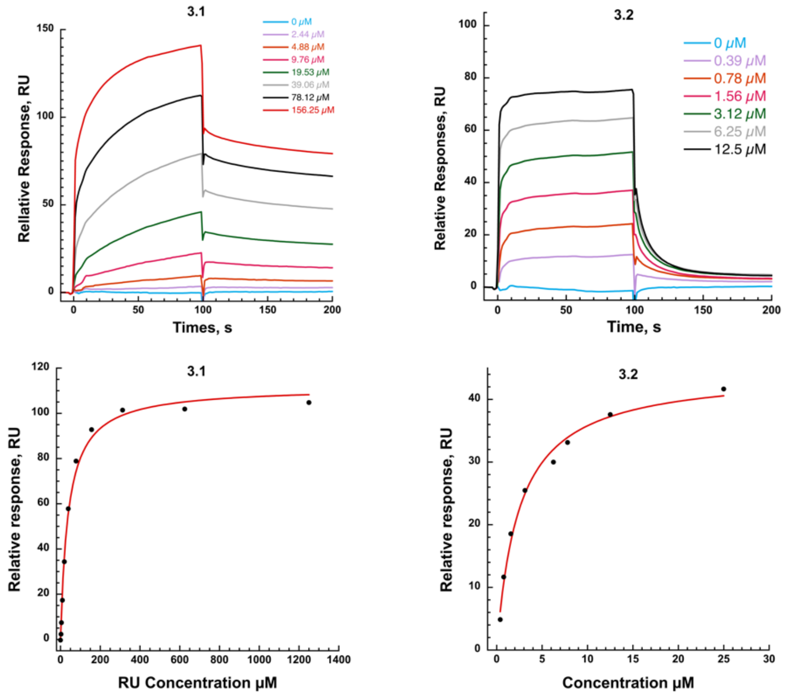

Compound **1.6**	Compound **2.6**	Compound **3.6**

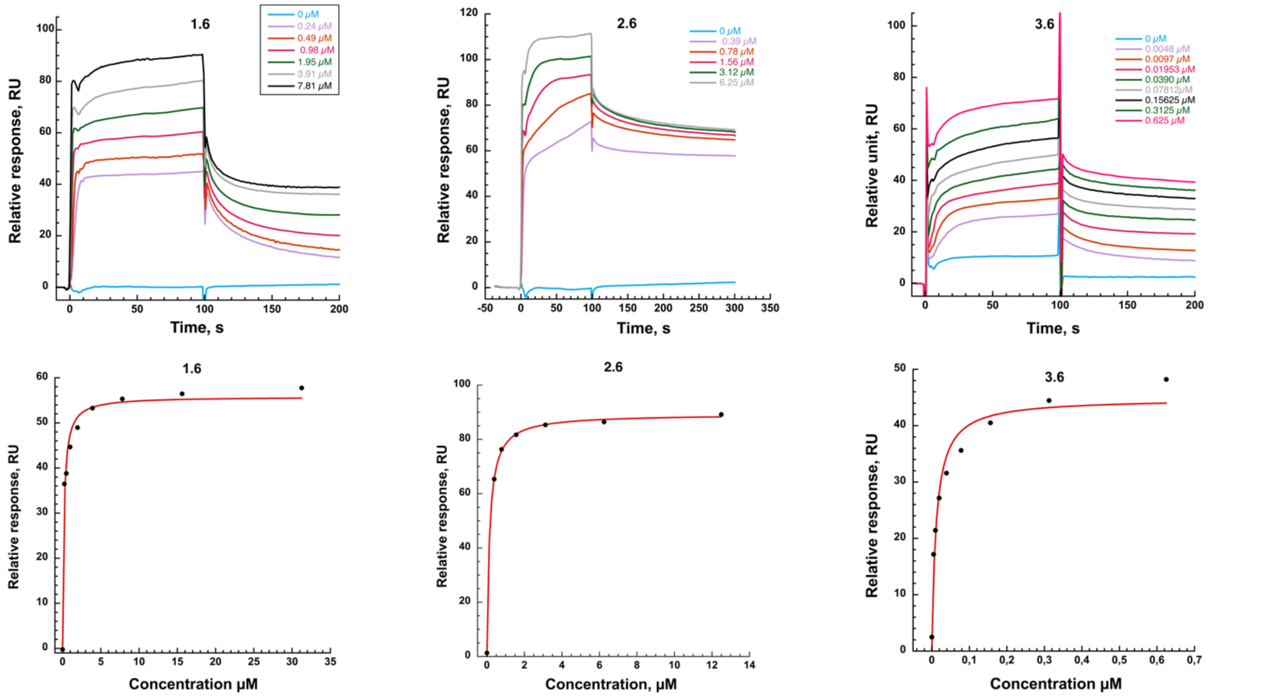

Figure S1: Binding analysis of "rod-like" dendrimers over a DC-SIGN oriented surface. For each compound, the upper panel represents the reference surface corrected sensorgram showing the binding of indicated compound over a DC-SIGN S-ECD surface. The lower panel illustrated the binding responses plotted against their respecting concentrations and the curve was fitted by a *steady state affinity* model. The binding responses are measured just after the end of association, at the beginning of the dissociation phase.

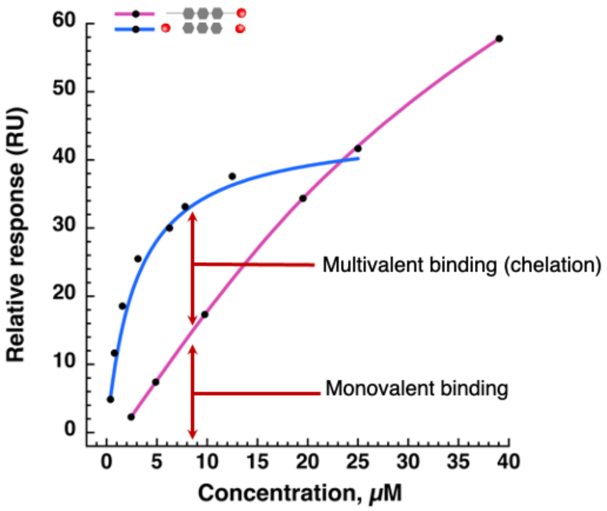

Figure S2: Qualitative comparison of association binding of compound **3.1** (magenta line) and **3.2** (blue line) over a DC-SIGN S-ECD surface in the range of 0-40 μM.

As illustrated on Figure S2, a comparative analysis of **3.1** and **3.2** binding was done over the same DC-SIGN oriented surface. From 0 to 25 μM, the divalent and monovalent "rod-like" dendrimers have a relative response significantly different. Compound **3.2** reaches to bind tightly the surface at low concentrations with higher relative responses compared to its monovalent counterpart **3.1**. Starting from 25 μM of compound **3.2** reaches a R_{max} between 40 to 50 RU while 3.1 can continue to bind following a hyperbolic curve. 3.1 is binding up to a level around 100 RU (compare entire titration curve for 3.1 and 3.2 in Figure S1). 3.1 has only one ligand per compound and thus can binds two times more compounds onto the surface while each **3.2** will block two sites. This graph is supplementary evidence of the multivalent binding, here chelate binding mode for **3.2**, that occur over a DC-SIGN oriented surface.

3. Isothermal titration calorimetric analysis

ITC experiments were performed at 25°C using TA Instrument Nano Isothermal Titration Calorimeter Low volume (Nano ITC LV). Compound **3.6**, **3.2** and DC-SIGN ECD were prepared in 25 mM Tris pH 8; 150 mM NaCl, 4 mM $CaCl_2$, 4 % DMSO. Compounds (30 μM of **3.6** or 375 μM of **3.2**) were stepwise injected (1.03 μL) to 190 μL of DC-SIGN ECD tetramer (respectively, 30 μM or 48.75 μM) in the cell. The blank titrations (compounds to buffer) were done for subtraction of dilution heat from the integrated data. A one-site binding model was fit to the data (nanoAnalyse 2.20 TA), yielding association constants (K_A) and binding enthalpies (ΔH).

The free energy changes (ΔG) and entropy (ΔS) were calculated using equation (2):

$$\Delta G = \Delta H - T\Delta S = -RT \ln K_A. \quad (2)$$

where T is the absolute temperature and R equal to 8.314 $J.mol^{-1}.K^{-1}$

4. Fluorescence polarization (FP) assay

Direct binding assays using fluorescence polarization equilibrium measurements were performed on multifunctional microplate reader CLARIOstar (BMG LABTECH) in 96 well plates (GREINER 96, F-BOTTOM no binding) with 150 µL of the assay solution per well. 360-20 nm excitation and 450-10 nm emission filters were used for the assays. In the FP saturation binding experiments, in triplicates, 5 µM of DC-SIGN tetramer were mixed with 400 nM of **3.6** and a serial dilution by a factor 2 was made with the buffer containing 400 nM of **3.6,** 25 mM Tris pH 8, 150 mM NaCl, 4 mM $CaCl_2$, 4% DMSO. The blank buffer is 25 mM Tris pH 8, 150 mM NaCl, 4 mM $CaCl_2$, 4% DMSO. Data was analyzed by Mars data analysis and the FP values were plotted against the DC-SIGN tetramer concentrations. EC_{50} was obtained using the *4-parameter logistic* equation.

5. Analytical Ultracentrifugation analysis

Experiments were performed using Beckman XL-I analytical ultracentrifuge with AN-60 and AN-50 TI rotor (Beckman instruments), at 20°C and 42 000 rpm, using 100 µl and 52 µl samples, loaded into the two-channel 3 and 1.5 mm path-length centerpieces with sapphire windows (Nanolytics). The absorbance at 280, 250 and 233 nm was monitored every 7 or 16 min for the set experiments, respectively, with a 30 µm radial step size.

The samples were prepared in buffer A (25 mM Tris pH 8, 150 mM NaCl, 4 mM $CaCl_2$, 4% DMSO). Experiments were done with samples contained 4 µM DC-SIGN ECD tetramer alone or with compound **3.6** at different concentrations (0.005, 0,05, 0.5, 2, 4, 6, 8 µM). The MW and partial specific volume of DC-SIGN monomer were estimated from amino acid composition using SEDNTERP software (Daemon version 20120828 Beta) and resulted to 38.7 kDa and 0.73 $mL.g^{-1}$, respectively.
SV profiles were analyzed using the continuous c(s) distribution analysis embedded in the SEDFIT software (version 15.01b).
The c(s) analysis was performed considering 200 particles, and fitting the frictional ratio, f/f0, the meniscus and the bottom of the cell. For the regularization, a 0.68 confidence level was used. All s-values were corrected for solvent density (1.005 g/mL) and viscosity (1.024 cp), obtained using SEDNTERP software (Daemon version 20120828 Beta), and thus are given as S_{20w}-values.

6. Molecular Modeling

6.1. Compound building and parametrization.

3D models of **1.6, 2.6** and **3.6** compounds were constructed based on coordinates from our previous work [2]. We built them in modular fashion to simplify parametrization and modelling. We defined several types of fragments (Figure S3), modeled the missing parts including hydrogens in PyMol (Molecular Graphics System,

Version 1.7.4. Schrödinger, LLC), capped the open valences by hydrogens, assigned GAFF2 parameters [6,7] and calculated AM1-BCC RESP partial charges [8,9]. Thereafter, the linking hydrogen atoms were removed, the remaining charge dispersed over the fragment and the fragments joined. All the parameters are available at section 6.1 of ref[10].

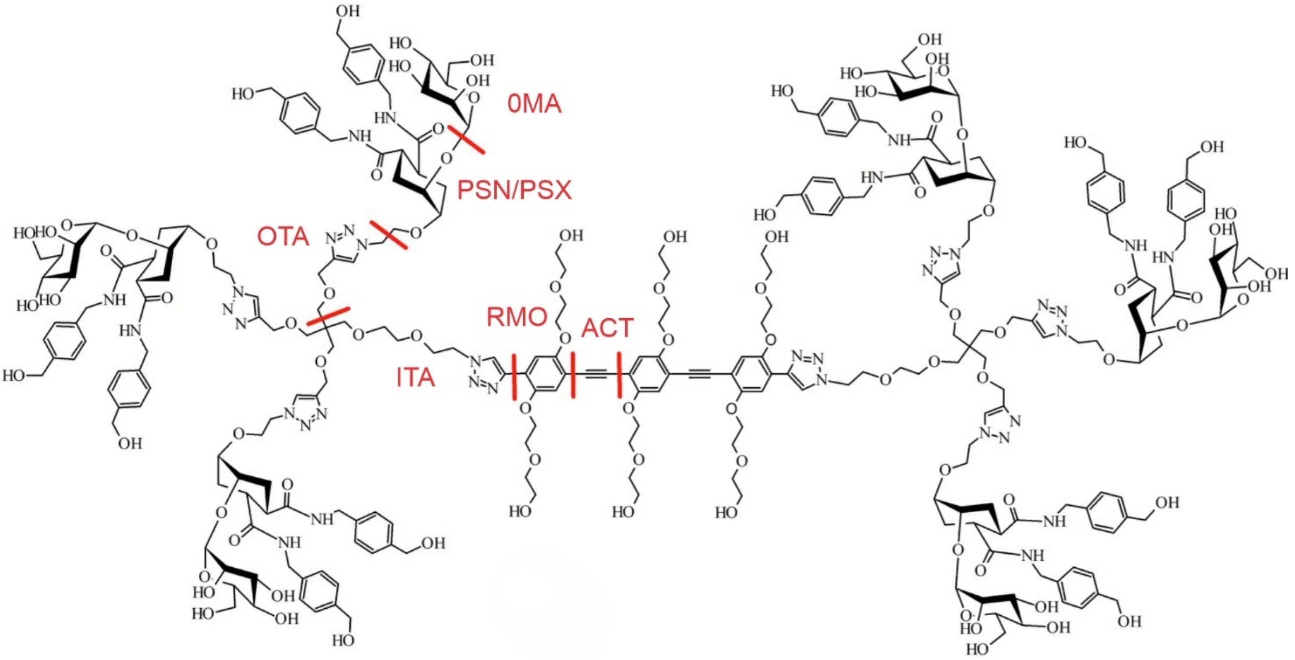

Figure S3. Chemical structure of compound **3.6**. Fragment names and delineation are in red.

6.2. High-temperature MD of compounds in implicit solvent

1.6, 2.6 and **3.6** compounds were subjected to 100 ns MD simulations at high temperature of 1000 K in generalized Born implicit solvent model (IGB=7) [11] in AMBER18 [12] to accelerate conformational sampling. The maximal distances that can be bridged by the compounds were obtained from the nine distances between OBG atoms of PSN/PSX residues measured between three spearheads from left- and right-side of the compounds.

6.3. Binding Modes of Compounds to Static DC-SIGN Tetramer

The DC-SIGN tetramer model fitted in the SAXS envelope was obtained from our previous work. [13] Four copies of chain A of the crystal structure of DC-SIGN monomer with pseudo-dimannoside ligand bound (PDB: 6GHV) [14] were transposed onto the tetramer and the 2-(4-amino-methyl)-triazole was changed into hydroxy group. To relieve of clashes in the resulting model, the N-terminal residues 253 to 259 were discarded and the system was subsequently optimized (the clashing Arg309 alone first) using ff14SB force field [15] for the protein, GAFF2 for the ligands (6, 7) and scaled charge parameters for calcium (ZC moiety; Electronic Continuum Correction with Rescaling; ECCR).[17] All the parameters are available at section 6.3. of ref [10]. One mannose unit of the tetramer DC-SIGN/ligand complex and of the optimized compounds **1.6, 2.6.** or **3.6** were geometrically aligned using in-house scripts. The distal-end pseudo-mannose unit was brought to the bonding distance of the DC-SIGN-bound mannose by minimization in generalized Born implicit solvent model (igb=5) [18] using harmonic distance restraints ranging from 1-10 kcal/mol/Å^2. In the resulting *Side, Diag1* and *Diag2*

models (PDB files at section 6.3. of ref [10]), the whole ligand was optimized, holding the protein, the calcium ions and the mannose units frozen.

6.4. Modeling DC-SIGN Tetramer Including Neck

DC-SIGN homology models consisting of four CRDs and two C-terminal ECD repeats were built using Modeller, version 10. [19] The crystal structures of a monomeric CRD fragment from DC-SIGN (PDB: 1K9I_D) and a tetrameric coiled coil fragment from DC-SIGNR (PDB: 3JQH) were identified as suitable templates for modelling following a BLASTp of the Protein Data Bank (PDB) using the canonical DC-SIGN sequence, obtained from UniProt (www.uniprot.org). The ECD fragment covered 100% of the ECD sequence, with an identity of 93%. The CRD fragment had an identity of 100% compared with the sequence, but only covered 84% of the sequence. This was attributed to 20 amino acids of the C-terminal tail which were not present in any DC-SIGN crystal structure.

Since there were no overlapping regions of the CRD and ECD crystal structures, the crystal structure of the DC-SIGNR CRD (PDB: 1XAR_A), which includes the final C-terminal half of one neck repeat, was used as a guide to generate alignment of the crystal structures using PyMOL (Molecular Graphics System, Version 1.7.4. Schrödinger, LLC).

An ensemble of 700 homology models were created to sample a broad range of CRD arrangements. Models were selected by measuring the angles between alpha helices in the CRDs, and between alpha helices in the CRDs and the neck. In addition, distances between the C-alpha atom of the aspartate residue in the CRD (which coordinates Ca^{2+}) were measured. The aforementioned angles and distances of the selected models were all within one standard deviation of those observed in the SAXS model and were ranked, using the SOAP protein potential in Modeller, in the top 10% of the ensemble.

Each of the selected models were used as templates in the generation of 10 more homology models, which would include the C-terminal tail modelled from scratch by Modeller, and the highest scoring models derived from each template were simulated for 10 ns in implicit solvent using CHARMM[20].

One model was selected from the simulations, based on its stability measured by RMSD. The C-terminal tails in this model interacted with either the ECD or an adjacent CRD. This model was further used in production MD simulations.

6.5. Dynamics of DC-SIGN Tetramer Including Neck

AMBER force field ff14SB [15] parameters were assigned to the protein, except aspartates 114, 149, 160, 161 and glutamates 118, 141, 147, 148 for whose side chains reduced charges were used to be compatible with ECC charges on Ca.[17] The protein was immersed in an octahedral box of explicit TIP4P-2005 water [21] molecules, extending up to 12 Å from the protein. Scaled-charge K^+ and Cl^- counterions were added to neutralize the charge and match to a final concentration of 0.15 M. All the parameters are available at section 6.5 of ref [10]. Stepwise relaxation was thus carried out according to the published protocols [22,23], except that the length of the production MD was 2μs. The 6 Ca···Ca distances (4 *Side*, 2 *Diag*) were monitored and became

relatively stable from 500 ns onward. The analyses were and thus carried out on the last 1.5 μs of the trajectory (available at section 6.5 of ref [10])

7. Considerations on clustering binding modes

In a geometrically simplified view, two structurally possible extreme cases of DC-SIGN tetramer arrangements can be envisaged – rhombus and square. When closely positioned on a surface, this allows packing of three rhombi or four squares, respectively (Fig. S4). Using the distances from the static DC-SIGN tetramer model, each of the three compounds **1.6, 2.6,** and **3.6** could reach to four CRDs via clustering mode (on top of two via chelation) in the rhombus case. The longest separation of two CRDs characterized by the *Diag2* Ca···Ca distance would be 69.3 Å as follows from Eq. 2 and *Side* distance of 40 Å (Tab. 3).

$$Diag2 = Side * \sqrt{(2 + 2\cos(60°))} \text{.... Eq. 2,}$$

i.e. it would exceed the maximal distance attainable by the **3.6** compound of 62.7 Å (see above) and thus it could not be effectively bridged (Fig. S4A). The same binding characteristics for all three compounds as well as the inability of compound **3.6** to bridge the *Diag2* chelating mode contradict the experimental avidity differences (Table 1). In that case clustering could not be the source for the differential avidity observed and thus it excludes the rhombus arrangement.

On the contrary, the square arrangement (Fig. S4B) offers the same number of attainable binding sites only for compounds **1.6** and **2.6** as both fall short (49.6 and 56.1 Å, respectively) of the *Diag* distance of 56.6 Å which comes from Eq. 3 and *Side* distance of 40 Å (Tab. 3).

$$Diag = \sqrt{(2 * Side^2)} \text{...Eq. 3,}$$

In contrast, the compound **3.6** adds three more binding sites in three neighboring DC-SIGN tetramers (on top of one additional site across the *Diag* distance in the chelating mode, described in the main text). This difference between the binding of compounds **1.6, 2.6** on one hand and **3.6** on the other hand could contribute to the avidity boosting of the latter compound by a combination of chelating and clustering binding modes, if DC-SIGN tetramers were in the square arrangement (Fig. S4B).

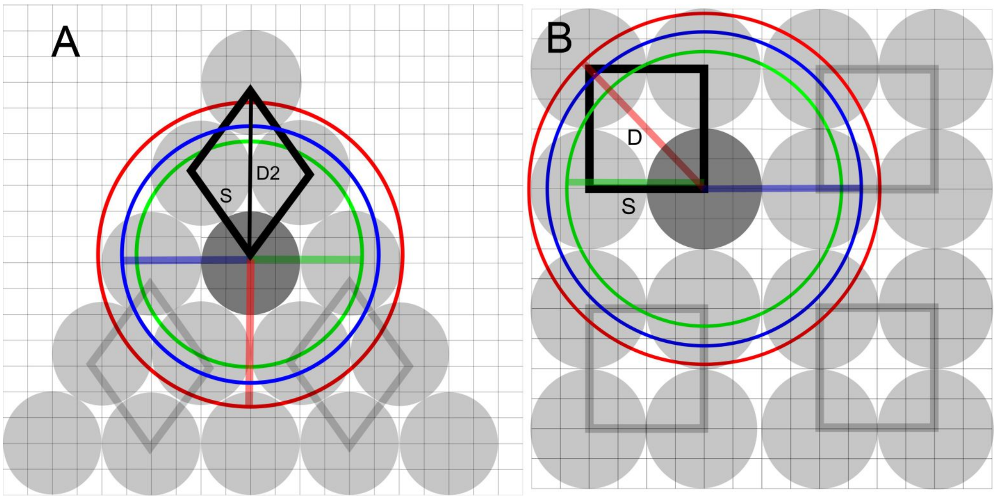

Fig. S4. Models of structurally possible arrangements of DC-SIGN tetramers. (A) rhombus, (B) square. Each vertex of a rhombus or square represents a CRD. Grey circles represent DC-SIGN monomers, in dark gray the monomer where one mannose unit of multivalent compounds binds. The radius of farthest accessible Ca^{2+} sites (maximal Ca···Ca distance) for compounds **1.6, 2.6** and **3.6** binding are shown as green, blue and red circles, respectively. *Side* (S) and *Diagonal* (D, D2) distances in the tetramers are shown.

To assess whether such a highly dense array of DC-SIGN lectin receptors as in Fig. S4B would be probable in the context of a sensor chip, we need to consider how oriented surfaces of DC-SIGN are generated experimentally (Fig. 1D). The first layer of chip functionalization is streptactin onto which DC-SIGN ECDs are captured via their N-terminal Streptag II. The known experimental density of Streptactin suggests that it covers the chip surface quite sparsely. Moreover, more than one of the four Streptag II at the N-termini of a single DC-SIGN ECD tetramer may bind to the available sites of a given streptavidin, thus further diminishing the surface concentration of DC-SIGN ECD. It is more probable that only one, maximum two, DC-SIGN tetramers are present per Streptactin. Then, only two DC-SIGN tetramers per Streptactin would be considered to produce internal clustering modes. Thus, the theoretical dense arrays of CRDs, as presented above (Figure S4), represent extreme largely improbably model cases. This underlies the essential role of chelation in the avidity boost observed for such rod-base compounds. We should also mention that the situation will be totally different for big entities like a virus presenting glycosylated envelope protein, as for the SARS-CoV-2, for instance. In that case, due to its large size and multiple spike glycoproteins, clustering interactions involving several lectin receptors will be more easily attainable.

References

(1) Varga, N.; Sutkeviciute, I.; Guzzi, C.; McGeagh, J.; Petit-Haertlein, I.; Gugliotta, S.; Weiser, J.; Angulo, J.; Fieschi, F.; Bernardi, A. Selective Targeting of Dendritic Cell-Specific Intercellular Adhesion Molecule-3-Grabbing Nonintegrin (DC-SIGN) with Mannose-Based Glycomimetics: Synthesis and Interaction Studies of Bis(Benzylamide) Derivatives of a Pseudomannobioside. *Chem. - Eur. J.* **2013**, *19* (15), 4786–4797. https://doi.org/10.1002/chem.201202764.

(2) Ordanini, S.; Varga, N.; Porkolab, V.; Thépaut, M.; Belvisi, L.; Bertaglia, A.; Palmioli, A.; Berzi, A.; Trabattoni, D.; Clerici, M.; Fieschi, F.; Bernardi, A. Designing Nanomolar Antagonists of DC-SIGN-

Mediated HIV Infection: Ligand Presentation Using Molecular Rods. *Chem. Commun.* **2015**, *51* (18), 3816–3819. https://doi.org/10.1039/c4cc09709b.

(3) Berzi, A.; Ordanini, S.; Joosten, B.; Trabattoni, D.; Cambi, A.; Bernardi, A.; Clerici, M. Pseudo-Mannosylated DC-SIGN Ligands as Immunomodulants. *Sci. Rep.* **2016**, *6*, 35373. https://doi.org/10.1038/srep35373.

(4) Berzi, A.; Varga, N.; Sattin, S.; Antonazzo, P.; Biasin, M.; Cetin, I.; Trabattoni, D.; Bernardi, A.; Clerici, M. Pseudo-Mannosylated DC-SIGN Ligands as Potential Adjuvants for HIV Vaccines. *Viruses* **2014**, *6* (2), 391–403. https://doi.org/10.3390/v6020391.

(5) Porkolab, V.; Pifferi, C.; Sutkeviciute, I.; Ordanini, S.; Taouai, M.; Thépaut, M.; Vivès, C.; Benazza, M.; Bernardi, A.; Renaudet, O.; Fieschi, F. Development of C-Type Lectin-Oriented Surfaces for High Avidity Glycoconjugates: Towards Mimicking Multivalent Interactions on the Cell Surface. *Org. Biomol. Chem.* **2020**, *18* (25), 4763–4772. https://doi.org/10.1039/D0OB00781A.

(6) Wang, J.; Wolf, R. M.; Caldwell, J. W.; Kollman, P. A.; Case, D. A. Development and Testing of a General Amber Force Field. *J. Comput. Chem.* **2004**, *25* (9), 1157–1174. https://doi.org/10.1002/jcc.20035.

(7) Wang, J.; Wang, W.; Kollman, P. A.; Case, D. A. Automatic Atom Type and Bond Type Perception in Molecular Mechanical Calculations. *J. Mol. Graph. Model.* **2006**, *25* (2), 247–260. https://doi.org/10.1016/j.jmgm.2005.12.005.

(8) Jakalian, A.; Bush, B. L.; Jack, D. B.; Bayly, C. I. Fast, Efficient Generation of High-Quality Atomic Charges. AM1-BCC Model: I. Method. *J. Comput. Chem.* **2000**, *21* (2), 132–146. https://doi.org/10.1002/(SICI)1096-987X(20000130)21:2<132::AID-JCC5>3.0.CO;2-P.

(9) Jakalian, A.; Jack, D. B.; Bayly, C. I. Fast, Efficient Generation of High-Quality Atomic Charges. AM1-BCC Model: II. Parameterization and Validation. *J. Comput. Chem.* **2002**, *23* (16), 1623–1641. https://doi.org/10.1002/jcc.10128.

(10) Lepsik, M.; Saint John, A. DC-SIGN Lectin / Multivalent Glycomimetics: Parametrisation and Molecular Dynamics. Mendeley 2022. https://doi.org/10.17632/4TDW25K6MC.1.

(11) Mongan, J.; Simmerling, C.; McCammon, J. A.; Case, D. A.; Onufriev, A. Generalized Born Model with a Simple, Robust Molecular Volume Correction. *J. Chem. Theory Comput.* **2007**, *3* (1), 156–169. https://doi.org/10.1021/ct600085e.

(12) Case, D. A., Ben-Shalom, I. Y., Brozell, S. R., Cerutti, D.S., Cheatham, T.E., Cruzeiro, III, V.W.D., Darden, T.A., Duke, R.E., Ghoreishi, D., Gilson, M.K. et al. *AMBER 2018*; University of California, San Francisco., 2018.

(13) Reina, J. J.; Díaz, I.; Nieto, P. M.; Campillo, N. E.; Páez, J. A.; Tabarani, G.; Fieschi, F.; Rojo, J. Docking, synthesis, and NMR studies of mannosyl trisaccharide ligands for DC-SIGN lectin. *Org. Biomol. Chem.* **2008**, *6* (15), 2743–2754. https://doi.org/10.1039/b802144a.

(14) Medve, L.; Achilli, S.; Guzman-Caldentey, J.; Thépaut, M.; Senaldi, L.; Le Roy, A.; Sattin, S.; Ebel, C.; Vivès, C.; Martin-Santamaria, S.; Bernardi, A.; Fieschi, F. Enhancing Potency and Selectivity of a DC-SIGN Glycomimetic Ligand by Fragment-Based Design: Structural Basis. *Chem. - Eur. J.* **2019**, *25* (64), 14659–14668. https://doi.org/10.1002/chem.201903391.

(15) Maier, J. A.; Martinez, C.; Kasavajhala, K.; Wickstrom, L.; Hauser, K. E.; Simmerling, C. Ff14SB: Improving the Accuracy of Protein Side Chain and Backbone Parameters from Ff99SB. *J. Chem. Theory Comput.* **2015**, *11* (8), 3696–3713. https://doi.org/10.1021/acs.jctc.5b00255.

(16) Kirschner, K. N.; Yongye, A. B.; Tschampel, S. M.; González-Outeiriño, J.; Daniels, C. R.; Foley, B. L.; Woods, R. J. GLYCAM06: A Generalizable Biomolecular Force Field. Carbohydrates: GLYCAM06. *J. Comput. Chem.* **2008**, *29* (4), 622–655. https://doi.org/10.1002/jcc.20820.

(17) Martinek, T.; Duboué-Dijon, E.; Timr, Š.; Mason, P. E.; Baxová, K.; Fischer, H. E.; Schmidt, B.; Pluhařová, E.; Jungwirth, P. Calcium Ions in Aqueous Solutions: Accurate Force Field Description Aided by *Ab Initio* Molecular Dynamics and Neutron Scattering. *J. Chem. Phys.* **2018**, *148* (22), 222813. https://doi.org/10.1063/1.5006779.

(18) Onufriev, A.; Bashford, D.; Case, D. A. Exploring Protein Native States and Large-Scale Conformational Changes with a Modified Generalized Born Model. *Proteins Struct. Funct. Bioinforma.* **2004**, *55* (2), 383–394. https://doi.org/10.1002/prot.20033.

(19) Šali, A.; Blundell, T. L. Comparative Protein Modelling by Satisfaction of Spatial Restraints. *J. Mol. Biol.* **1993**, *234* (3), 779–815. https://doi.org/10.1006/jmbi.1993.1626.

(20) Brooks, B. R.; Brooks, C. L.; Mackerell, A. D.; Nilsson, L.; Petrella, R. J.; Roux, B.; Won, Y.; Archontis, G.; Bartels, C.; Boresch, S.; Caflisch, A.; Caves, L.; Cui, Q.; Dinner, A. R.; Feig, M.; Fischer, S.; Gao, J.; Hodoscek, M.; Im, W.; Kuczera, K.; Lazaridis, T.; Ma, J.; Ovchinnikov, V.; Paci, E.; Pastor, R. W.;

Post, C. B.; Pu, J. Z.; Schaefer, M.; Tidor, B.; Venable, R. M.; Woodcock, H. L.; Wu, X.; Yang, W.; York, D. M.; Karplus, M. CHARMM: The Biomolecular Simulation Program. *J. Comput. Chem.* **2009**, *30* (10), 1545–1614. https://doi.org/10.1002/jcc.21287.

(21) Abascal, J. L. F.; Vega, C. A General Purpose Model for the Condensed Phases of Water: TIP4P/2005. *J. Chem. Phys.* **2005**, *123* (23), 234505. https://doi.org/10.1063/1.2121687.

(22) Srb, P.; Svoboda, M.; Benda, L.; Lepšík, M.; Tarábek, J.; Šícha, V.; Grüner, B.; Grantz-Šašková, K.; Brynda, J.; Řezáčová, P.; Konvalinka, J.; Veverka, V. Capturing a Dynamically Interacting Inhibitor by Paramagnetic NMR Spectroscopy. *Phys. Chem. Chem. Phys.* **2019**, *21* (10), 5661–5673. https://doi.org/10.1039/C9CP00416E.

(23) Lepsik, M.; Sommer, R.; Kuhaudomlarp, S.; Lelimousin, M.; Paci, E.; Varrot, A.; Titz, A.; Imberty, A. Induction of Rare Conformation of Oligosaccharide by Binding to Calcium-Dependent Bacterial Lectin: X-Ray Crystallography and Modelling Study. *Eur. J. Med. Chem.* **2019**, *177*, 212–220. https://doi.org/10.1016/j.ejmech.2019.05.049.
