# Supplementary material for: Powerful Avidity with a Limited Valency for Virus-Attachment Blockers on DC-SIGN: Combining Chelation and Statistical Rebinding with Structural Plasticity of the Receptor
Source: ACS Cent Sci. 2023 Feb 20;9(4):709–18. doi: 10.1021/acscentsci.2c01136 (PMC10141607; doi:10.1021/acscentsci.2c01136)
Supplement: Supplementary file 4 — oc2c01136_si_004.pdf [file oc2c01136_si_004.pdf]

oc-2022-01136k.R1

Name: Peer Review Information for "Powerful avidity with a limited valency for virus-attachment blockers on DC-SIGN: Combining chelation and statistical rebinding with structural plasticity of the receptor"

First Round of Reviewer Comments

Reviewer: 1

Comments to the Author

The manuscript “Powerful avidity with a limited valency for virus-attachment blockers on DC SIGN: Combining chelation and statistical rebinding with structural plasticity of the receptor” by Fieschi and coworkers provides relevant information about the topic of multivalency and the different binding modes responsible to the increment of avidity in multivalent interactions. For this aim, authors has used as a proof of concept a particular example. As they mentioned, there is a plethora of examples describing carbohydrate multivalent compounds but a lack of information respect to the factors contributing to increase the binding affinity. In this elegant approach, authors use different techniques to obtain very important evidences about this type of processes. These findings can be extrapolated to others cases and should be very important for the scientific community working in the field of multivalent interactions. Moreover, this information will pave the way for a more rational and effective design of multivalent systems taking into account the factors that govern the avidity of the recognition process. This information could be used to prepare interesting chemical tools or even to develop new compounds to be used as drugs in different applications.

However, some corrections should be done to improve the quality of the manuscript and some relevant data should be included to support some conclusions. In particular

- Pag 6: the sentence “IC50 always underestimate affinity with respect to KD, suggesting that real affinity difference between 1 and 3.1 is probably lower than the factor of 6” cannot be supported by the data presented in Table 2, KD and IC50 are missed for compound 1 and 3.1, respectively. Also, the KD for compound 3.2-long is not in the table and it should be very relevant to see the impact of flexibility (and entropy) in the avidity for comparison to compound 3.2

- Pag 7. Previously, it is mentioned that the IC50 data underestimate affinity but here “This occurs despite the entropy loss caused by the presence of a flexible linker in 3.6, which we can estimate contributes by a negative factor of 2, as judged by comparison of the inhibition data for 3.2 and the 3.2-long control” it is used for comparison purposes, maybe because the KD is missed for one of the compounds (3.2-long)

- The style of the references should be revised carefully (journals in many of them are not abbreviated correctly)

Minor changes:

In table 2, it should be indicated entry numbers

In the Title of the manuscript, the hyphen in DC-SIGN is missed

Colors used in Figure 2D should be changed for clarity

All these issues should be revised and corrected before the manuscript will be accepted for publication

Reviewer: 2

Comments to the Author

The manuscript submitted by Prof Fieschi et al. reports on the use of previously reported lectin ligands and their binding properties towards DC-SIGN. Although the synthesis of the compounds has been already reported by the authors, the present study represents an intensive and careful study of the binding properties of each glycoconjugate with a very good understanding of the parameters governing the multivalent carbohydrate-lectin interactions such as chelate effect or statistical rebinding for instance. Therefore, even though the molecules studied here have been largely rpeorted before, the complete study of the binding properties toward DC-SIGN presented here can make of this manuscript one of the leaidng articles in the field characterizing exactly, precisely and with reliable experimental data the topic of multivalent carbohydrate interactions.

I therefore recommend publication with the revisions listed below.

Page 3, line 13: references 3-6,7 should appear as 3-7 ?

In reference 21 about multivalent lectin ligands, please include Chem Rev 2015, 115, 525-561

Some numbers of compounds (3.6, 2.6 or others) are not always "bold" layout in the main text and supp info.

The authors have used a KDapp value for the ITC data (pages 7-8, and Table 2). ITC should provide a "real" KD value, not an apparent one. If this is a simple typing mistake, then a quick correction should do it. If not, then why using the KDapp should me discussed at the beginning of this section.

Legend of Figure 3: Title could read "Isothermal titration microcalorimetry of compound..."

and then in title of Table 2: "Thermodynamic parameters of multivalent binding between between compounds 3.2 and 3.6 toward DC-SIGN as measured by ITC"

Page 13, line 4: Please define FP

Page 13, line 50: replace "sugar" with "carbohydrate"

Page 14, line 12: Please include reference 21 with reference 20 about multivalent glycoclusters targeting DC-SIGN.

Formatting of references is not correct regarding the journal abbreviations.

The spelling of Dr Sutkeviciute is wrong in reference 14 (and maybe others)

Supporting information: Please provide only 4 digits for the HRMS data of compound 3.1

Author's Response to Peer Review Comments:

Pr. Franck Fieschi
Équipe Membranes et Immunité,
Groupe Membrane et Pathogènes,
Institut de Biologie Structurale,
71 Avenue des martyrs,
CS 10090
38044 Grenoble CEDEX 9

Grenoble 20th Nov. 2022

Answer to referees

Reviewer: 1

Recommendation: Reconsider after major revisions noted.

Comments:
The manuscript "Powerful avidity with a limited valency for virus-attachment blockers on DC SIGN: Combining chelation and statistical rebinding with structural plasticity of the receptor" by Fieschi and coworkers provides relevant information about the topic of multivalency and the different binding modes responsible to the increment of avidity in multivalent interactions. For this aim, authors has used as a proof of concept a particular example. As they mentioned, there is a plethora of examples describing carbohydrate multivalent compounds but a lack of information respect to the factors contributing to increase the binding affinity. In this elegant approach, authors use different techniques to obtain very important evidences about this type of processes. These findings can be extrapolated to others cases and should be very important for the scientific community working in the field of multivalent interactions. Moreover, this information will pave the way for a more rational and effective design of multivalent systems taking into account the factors that govern the avidity of the recognition process. This information could be used to prepare interesting chemical tools or even to develop new compounds to be used as drugs in different applications.

We warmly thank referee 1 for his thorough review of the manuscript, his strong interest in our work and his really positive evaluation (Especially the Top 1% significance to chemistry researchers in this and related fields). It is a real encouragement in our efforts to characterize the fundamental mechanisms of avidity.

However, some corrections should be done to improve the quality of the manuscript and some relevant data should be included to support some conclusions. In particular

- Pag 6: the sentence "IC50 always underestimate affinity with respect to KD, suggesting that real affinity difference between 1 and 3.1 is probably lower than the factor of 6" cannot be supported by the data presented in Table 2,

We believe that referee1 is making reference to data from Table 1 and not Table 2.

The referee is perfectly right that this sentence is not supported by our data. However, we did not state that it was the case, we just stated a general rule, supported by the Cheng-Prusoff relationship. In the case of a competitive inhibition assay, used here for several compounds, such as 1, 3.2 and 3.6, the IC_{50} can be directly related to the inhibition constant K_i (which is in fact K_d for compound used as binding inhibitors) thanks to the Cheng-Prusoff relationship:

$$\mathbf{IC_{50} = K_i\ (1 + [S]/K_d)} \qquad \text{equation 1}$$

Here, in our SPR competitive assay, [S] is the glycoconjugate “concentration” on the surface, K_d is the dissociation constant of DC-SIGN from the glycoconjugate functionalized surface. K_i is K_d for the compounds used as binding inhibitor in the soluble phase and IC_{50} is the concentration of ligand needed to inhibit at 50 % of the reporter interaction in our assay.

Here IC_{50} is measured in the experiment, K_d of DC-SIGN for the surface can be known from titration experiment (and it is around 5 µM in our case), [S] is difficult to evaluate properly (it depends simultaneously on the density of glycoconjugates bound on the surfaces as well as on the level of glycosylation per glycoconjugate and finally here the ligands for DC-SIGN are more on a “surface” rather than in a volume). Thus, without a reliable evaluation of [S], it is not possible to calculate Ki from the IC50.
However, **from equation 1, we can algebraically state that IC_{50} will always be larger for a competitive inhibitor than its theoretical Ki (= Kd).** In a limiting case where [S] will be very high with respect to Kd, IC_{50} will tend to be only slightly larger than Ki.

Then, if the IC_{50} of a compound is used as a way to evaluate the affinity instead of its real K_d, and the IC_{50} has a higher value than the K_d, the affinity will be underestimated.

So when we wrote “IC_{50} always underestimate affinity with respect to K_d, suggesting that real affinity difference between **1** and **3.1** is probably lower than the factor of 6” **it was not based *on our data* but was simply a general statement coming from what is known from theory** (relationship between IC_{50} and Ki (K_d) - Cheng-Prusoff equation in the context of competition experiments).

To make it more clear we propose to replace the sentence :

IC50 always underestimate affinity with respect to KD, suggesting that…

By the following sentences and the addition of a reference to the Cheng-Prussoff treatment for competition assay.

As shown by the Cheng-Prussoff equation, the IC50 value of a competitive inhibitor is always higher than its K_D.[34] Thus, the affinity is often underestimated with the IC50, suggesting that…

Ref34 added is : Cheng Y. and WH. Prusoff, Biochem. Pharmacol. (1973) 22, 3099-3108)

KD and IC50 are missed for compound 1 and 3.1, respectively. Also, the KD for compound 3.2-long is not in the table and it should be very relevant to see the impact of flexibility (and entropy) in the avidity for comparison to compound 3.2.

Regarding the K_d for compound **1**, titration has been initially tested on an oriented surface to get a K_d. We have not been able to obtain reliable data and that is why for 1, as for monovalent ligand, we determined its IC_{50} by competition. Indeed, for this particular compound we combine low affinity (in the 10^{-4} concentration range which is close to the limit of measurement for SPR) with low molecular weight. Quality of the data for a direct K_d determination was not sufficient enough (high level of background).

Regarding compound **3.2 long**, we report here its IC50 from our previous work. Its Kd has not been determined using the new test with DC-SIGN oriented surface because this particular compound was no more available and it has not been resynthesized for these studies. However, by comparing its IC_{50} with **3.2**, it is clear that there is not a significant difference in affinity (it may be slightly worse).

- Pag 7. Previously, it is mentioned that the IC50 data underestimate affinity but here "This occurs despite the entropy loss caused by the presence of a flexible linker in 3.6, which we can estimate contributes by a negative factor of 2, as judged by comparison of the inhibition data for 3.2 and the 3.2-long control" it is used for comparison purposes, maybe because the KD is missed for one of the compounds (3.2-long).

The referee is right. It is because we do not have the K_d for one of the two compounds, then we prefer to compare their activity using their IC_{50}, that were obtained for both in the same set of experiments. Even though IC50 is not exactly K_d, it is more relevant to compare compounds by their IC_{50} obtained in analogous conditions, rather than comparing the IC_{50} of one with the K_d of the other. The absolute values of affinity might be underestimated with IC_{50} BUT the relative comparison between compounds is fully respected (there is strict proportionality between IC_{50} and K_d, see Cheng-Prusoff equation in the answer above). So, it is perfectly relevant to proceed that way.

- The style of the references should be revised carefully (journals in many of them are not abbreviated correctly).

Problem of references format has been solved and corrected.

Minor changes:
In table 2, it should be indicated entry numbers.

We do not understand the changes asked here by referee 1 since there is currently entry numbers for corresponding compounds (3.2 and 3.6). Sorry not to be able to answer maybe more adequately.

In the Title of the manuscript, the hyphen in DC-SIGN is missed.

Yes, thank you for noticing it. This is corrected in the new submission.

Colors used in Figure 2D should be changed for clarity

It has been changed as requested.

All these issues should be revised and corrected before the manuscript will be accepted for publication.

We hope that our answers meet satisfaction of the referee 1.

Additional Questions:
Quality of experimental data, technical rigor: Top 5%
Significance to chemistry researchers in this and related fields: Top 1%
Broad interest to other researchers: Top 5%
Novelty: Top 5%
Is this research study suitable for media coverage or a First Reactions (a News & Views piece in the journal)?: Yes.

Reviewer: 2

Recommendation: Publish in ACS Central Science after minor revisions noted.

Comments:
The manuscript submitted by Prof Fieschi et al. reports on the use of previously reported lectin ligands and their binding properties towards DC-SIGN. Although the synthesis of the compounds has been already reported by the authors, the present study represents an intensive and careful study of the binding properties of each glycoconjugate with a very good understanding of the parameters governing the multivalent carbohydrate-lectin interactions such as chelate effect or statistical rebinding for instance. Therefore, even though the molecules studied here have been largely reported before, the complete study

of the binding properties toward DC-SIGN presented here can make of this manuscript one of the leading articles in the field characterizing exactly, precisely and with reliable experimental data the topic of multivalent carbohydrate interactions.
I therefore recommend publication with the revisions listed below.

We feel very honored by the comments from referee2 and thank him warmly for his very kind comments on our *work ("one of the leading articles in the field"). This* is really a great encouragement for us and gives us a fantastic boost to continue our studies

Page 3, line 13: references 3-6,7 should appear as 3-7 ?

This has been corrected. Thanks for noticing this.

In reference 21 about multivalent lectin ligands, please include Chem Rev 2015, 115, 525-561.

The reference has been added.

Some numbers of compounds (3.6, 2.6 or others) are not always "bold" layout in the main text and supp info.

We have strived to correct all of them now in the main text as well as in the supporting information.

The authors have used a KDapp value for the ITC data (pages 7-8, and Table 2). ITC should provide a "real" KD value, not an apparent one. If this is a simple typing mistake, then a quick correction should do it. If not, then why using the KDapp should be discussed at the beginning of this section.

We are using K_{dapp}, and not K_{d} because it is related to a multivalent interaction and not to a unitary 1:1 interaction.
1) The "K_{d}" observed is then more relevant to an avidity than to an affinity and is the result of several cumulative unitary-binding events for which it is not possible to deconvolute their individual affinity contribution to the global avidity.
2) Moreover, this global avidity evaluated by ITC, in addition to be composed from several unitary interactions, also results from several multivalent modalities. Thus, here the global K_{d} value determined by ITC does not quantify one unique binding mode but a population of binding modes.
For these two reasons (avidity vs affinity and multiple avidity modes) we believe that it is more correct to use the global term "K_{dapp}" and not "K_{d}" that usually refers to a well-defined 1:1 interaction.
Using Kdapp instead of $\mathrm{K_d}$ here allows the reader not to forget that the value reported is resulting from a complex binding phenomenon.

Thus as asked by the referee, in order to justify the use of Kdapp instead of Kd, we added at the beginning of this section the following explanation.

"Usually, ITC should provide, among other parameters, a real K_D value; however, here we prefer to refer to a K_{Dapp}. Indeed, in these ITC experiments, we are not evaluating an affinity (a 1:1 unitary interaction, defined by K_D) but rather an avidity phenomenon resulting from cumulative unitary bonds (each of whose individual contributions to the overall avidity cannot be deconvoluted). Moreover, this avidity results from several multivalent modalities. Thus, the use of K_{Dapp}, instead of K_D, emphasizes that the values determined here are the result of a complex phenomenon."

Legend of Figure 3: Title could read "Isothermal titration microcalorimetry of compound..."
and then in title of Table 2: "Thermodynamic parameters of multivalent binding between between compounds 3.2 and 3.6 toward DC-SIGN as measured by ITC"

The title has been corrected as suggested.

Page 13, line 4: Please define FP

FP stands for fluorescence polarization. We have replaced the acronym by the full name.

Page 13, line 50: replace "sugar" with "carbohydrate

This has been corrected.

Page 14, line 12: Please include reference 21 with reference 20 about multivalent glycoclusters targeting DC-SIGN.

This has been done, as requested.

Formatting of references is not correct regarding the journal abbreviations.

The spelling of Dr Sutkeviciute is wrong in reference 14 (and maybe others)

Thank you, it has been corrected.

Supporting information: Please provide only 4 digits for the HRMS data of compound 3.1

Corrected

Additional Questions:
Quality of experimental data, technical rigor: High
Significance to chemistry researchers in this and related fields: High
Broad interest to other researchers: High
Novelty: Moderate
Is this research study suitable for media coverage or a First Reactions (a News & Views piece in the journal)?: No

Warm thanks to the two referees for their help in optimizing our manuscript

Pr. Franck Fieschi
Senior Member of the Institut Universitaire de France
Université Grenoble Alpes
Institut de Biologie Structurale
Group leader of Membrane & Pathogens
Team leader of Membrane & Immunity Team

oc-2022-01136k.R2

Name: Peer Review Information for "Powerful avidity with a limited valency for virus-attachment blockers on DC-SIGN: Combining chelation and statistical rebinding with structural plasticity of the receptor"

Second Round of Reviewer Comments

Reviewer: 2

Comments to the Author

The authors have addressed the changes required from thereview process and the manuscript has now reached the quality to be accepted for publication without changes.

Great work !!!

Reviewer: 1

Comments to the Author

Authors have answered satisfactory to all questions improving the quality of the manuscript. Only they should revised again some of the references to homogenize the citation style, check for instance references 12, 15, 16, 19, 24, 25, 30.......Except for this minor issue, the manuscript shoul be accepted for publication in its actual format.

Author's Response to Peer Review Comments:

Dear Editor,

As asked by referee 2, we have carefully corrected all the reference problem he cited.

This was the unique remaining corrections that was asked. You will have teh opportunity to control teh correction thanks to the annotated version fo the manuscript

As requested by Referee 2, we carefully corrected all of the reference problems he cited.

These were the only remaining corrections requested. You will have the opportunity to check these corrections with the annotated version of the corrected manuscript.

thank you for the excellent follow-up of our work,

best regards,

Franck Fieschi

oc-2022-01136k.R3

Name: Peer Review Information for "Powerful avidity with a limited valency for virus-attachment blockers on DC-SIGN: Combining chelation and statistical rebinding with structural plasticity of the receptor"

Third Round of Reviewer Comments

Reviewer: 1

Comments to the Author

Authors have addressed all the questions remaining for the improvement of the final version of the manuscript and therefor the paper can be accepted for publication

Reviewer: 2

Comments to the Author

The authors have adressed the changes and corrections required and the manuscript can be accepted for publication without further changes needed.

Author's Response to Peer Review Comments:

Dear editor,

Thanks for final acceptance of our work, TOC graphic and Synopsis has been added as asked. A Si paragraph has also been added at the end of the manuscript. I added also correct numbering to pages in the Supp.Info file and I also updated the reference format in the Supp. Info file as needed (using the ACS reference style).

Hope everything is fine now to proceed with proof soon.

In case you want to verify what has been done on the article and supp Info, i added annotated version of the new versions

Thanks for very much for everything,

Best regards,

Franck Fieschi
